# Distinct Roles of Non-Canonical Poly(A) Polymerases in RNA Metabolism

**DOI:** 10.1371/journal.pgen.1000555

**Published:** 2009-07-10

**Authors:** Salvatore San Paolo, Stepanka Vanacova, Luca Schenk, Tanja Scherrer, Diana Blank, Walter Keller, André P. Gerber

**Affiliations:** 1 Department of Cell Biology, Biozentrum, University of Basel, Basel, Switzerland; 2 National Center for Biomolecular Research, Masaryk University, Brno, Czech Republic; 3 Institute of Pharmaceutical Sciences, Department of Chemistry and Applied Biosciences, ETH Zürich, Zürich, Switzerland; The University of North Carolina at Chapel Hill, United States of America

## Abstract

Trf4p and Trf5p are non-canonical poly(A) polymerases and are part of the heteromeric protein complexes TRAMP4 and TRAMP5 that promote the degradation of aberrant and short-lived RNA substrates by interacting with the nuclear exosome. To assess the level of functional redundancy between the paralogous Trf4 and Trf5 proteins and to investigate the role of the Trf4-dependent polyadenylation *in vivo*, we used DNA microarrays to compare gene expression of the wild-type yeast strain of *S. cerevisiae* with either that of *trf4Δ* or *trf5Δ* mutant strains or the *trf4Δ* mutant expressing the polyadenylation-defective Trf4(DADA) protein. We found little overlap between the sets of transcripts with altered expression in the *trf4Δ* or the *trf5Δ* mutants, suggesting that Trf4p and Trf5p target distinct groups of RNAs for degradation. Surprisingly, most RNAs the expression of which was altered by the *trf4* deletion were restored to wild-type levels by overexpression of *TRF4(DADA)*, showing that the polyadenylation activity of Trf4p is dispensable *in vivo*. Apart from previously reported Trf4p and Trf5p target RNAs, this analysis along with *in vivo* cross-linking and RNA immunopurification-chip experiments revealed that both the TRAMP4 and the TRAMP5 complexes stimulate the degradation of spliced-out introns via a mechanism that is independent of the polyadenylation activity of Trf4p. In addition, we show that disruption of *trf4* causes severe shortening of telomeres suggesting that *TRF4* functions in the maintenance of telomere length. Finally, our study demonstrates that *TRF4*, the exosome, and *TRF5* participate in antisense RNA–mediated regulation of genes involved in phosphate metabolism. In conclusion, our results suggest that paralogous TRAMP complexes have distinct RNA selectivities with functional implications in RNA surveillance as well as other RNA–related processes. This indicates widespread and integrative functions of TRAMP complexes for the coordination of different gene expression regulatory processes.

## Author Summary

The discovery that most regions of the genome are actively transcribed into non-coding RNAs has dramatically increased interest in their function and regulation. Recent data from us and others have shed light on the molecular machinery that promotes the decay of such transcripts. In the yeast *S. cerevisiae*, Trf4p and Trf5p are alternative subunits of the so-called TRAMP complex, which degrades aberrant and short-lived RNAs. They add short poly(A) tails to their substrate RNAs that function as landing pads for exonucleases mediating RNA decay. Although alternate compositions of TRAMP complexes exist, the RNA substrate specificities and the processes controlled by them have not been determined. Applying a genome-wide approach, we describe overlapping yet distinct functional implications of different TRAMP complexes, and we demonstrate strong connections between RNA quality control and other RNA–related processes such as telomer length maintenance. Moreover, our study shows that the degradation of specific target RNAs is not strictly dependent on the polyadenylation activity of Trf proteins *in vivo*. These results suggest novel and integrative functions of TRAMP complexes for RNA regulation.

## Introduction

Gene expression in eukaryotes depends on highly complex mechanisms for production of mature RNA molecules. Precursors of mRNAs, ribosomal RNAs (rRNAs), transfer RNAs (tRNAs), small nucleolar RNAs (snoRNAs), and small nuclear RNA (snRNAs) undergo stepwise processing and maturation, which includes 5′-capping, splicing, 3′-polyadenylation, endo- and exonucleolytic trimming, and base modifications. All these processes are error-prone and thus, RNA maturation has to be monitored by nuclear and cytoplasmic RNA quality control pathways to remove potentially harmful aberrant RNAs [1],[2].

In the budding yeast *Saccharomyces cerevisiae*, nuclear RNA surveillance is mediated by the combined action of the Trf4/5-Air1/2-Mtr4 (TRAMP) complex and the exosome that promote rapid degradation of nonfunctional RNAs [3]–[10]. The TRAMP complex consists of either one of the two paralogous non-canonical poly(A) polymerases Trf4p and Trf5p forming TRAMP4 and TRAMP5 complexes, respectively, the RNA binding proteins Air1 or Air2, and the RNA helicase Mtr4p [5]–[7]. In contrast to the canonical poly(A) polymerase Pap1p, which adds long poly(A) tails to the 3′-end of mRNAs that facilitates nuclear RNA export and increases the stability and translation of messages [11],[12], the Trf proteins add short poly(A) tails to their substrate RNAs, which is assumed to trigger efficient decay of the RNAs by recruitment of the nuclear exosome complex [5]–[7].

Initially, Trf4 protein was identified as a key player in the surveillance and degradation of hypomodified initiator tRNA (tRNA_i_^Met^) [4]. Further studies revealed more widespread roles of TRAMP complexes to assist the exosome-mediated degradation and trimming of several types of non-coding RNAs (ncRNAs) including precursors of rRNAs, tRNAs, snoRNAs, snRNAs, and of aberrant pre-mRNAs that are defective in 3′ end cleavage, splicing, or export to the cytoplasm [1], [6]–[9], [13]–[17]. Many of these RNA substrates are part of ribonucleoprotein (RNP) complexes and pre-ribosomes suggesting that most if not all newly synthesized nuclear RNPs are subject to quality control by TRAMP and the exosome. Another major class of potential RNA targets for TRAMP complexes are the so-called ‘cryptic unstable transcripts’ (CUTs) [7]. CUTs are small, capped and fairly unstable transcripts that are expressed at such low levels that they can only be readily detected in nuclear RNA degradation mutants such as *rrp6Δ*. Originally detected in some intergenic regions (IGRs) [7], the recent systematic exploration of CUTs by RNA sequencing and tiling arrays suggests the existence of hundreds of CUTs that preferentially originate from nucleosome-free 5′ promotor regions, or from the 3′-ends of protein-coding genes [18],[19]. However, whether these CUTs have a biological role, or merely reflect transcriptional noise made from nucleosome-depleted regions is not known [18],[19].

Most studies investigating the functions of TRAMP complexes focused on TRAMP4 and much less is known about TRAMP5 [20],[21]. Trf4p and Trf5p share 56% amino-acid sequence similarity and loss of both poly(A) polymerases is lethal [22]. The conditional depletion of Trf5p in *trf4Δ* mutant cells increases the steady-state levels of specific RNAs, such as the 3′-extended forms of U14 snoRNA, the 23S pre-rRNA and the CUT *NEL025c* that accumulate in either single mutant indicating that Trf4p and Trf5p have at least partially overlapping substrate specificities *in vivo* [7],[15],[20],[23].

Besides RNA quality control and processing, Trf proteins may also participate in DNA-related processes. Originally, *TRF4* was identified in a screen for mutations that are synthetically lethal with *top1*, which encodes the DNA topoisomerase I [24]. A *top1 trf4-ts* double mutant was defective in several mitotic events, such as sister chromatid cohesion, chromosome condensation at the rDNA loci, and chromosome segregation [22], [24]–[26]. These defects were suppressed by overexpression of *TRF5* suggesting that both Trf4p and Trf5p have roles in DNA metabolism and heterochromatin formation [22]. Moreover, Trf4p as well as the orthologous protein Cid14 in *S. pombe* stimulate the RNA-mediated silencing of heterochromatic transcripts and control rDNA copy numbers [24], [27]–[32]. Hence, it was postulated that RNA-mediated recruitment of Trf4p and Trf5p may promote chromatin remodeling through regulation of histone modifying enzymes at specific chromatin loci [23].

Although the above mentioned studies revealed some substrates and functions for complexes containing Trf4 (TRAMP4) and Trf5 (TRAMP5), a comprehensive view of the substrate specificities and potential functional implications of the different TRAMP complexes is still lacking. We therefore wished to obtain a global picture of the RNA substrates that are regulated by the TRAMP4 and TRAMP5 complexes. To this end, we have used DNA microarrays to systematically map the RNA targets of Trf4p and Trf5. Surprisingly, we found that the different TRAMP complexes *per se* regulate only marginally overlapping sets of RNAs in the cell. Furthermore, the polyadenylation-defective form of Trf4p (Trf4p-DADA) suppressed most of the altered expression pattern as seen in the *trf4Δ* mutant cells suggesting that the TRAMP polyadenylation activity is not essential for RNA regulation. We further demonstrate that Trf4p and to a lower extent Trf5p promotes the degradation of a group of introns through an exosome-dependent but polyadenylation-independent mechanism. Moreover, Trf4p but not Trf5p stimulates RNA degradation mechanisms that are functionally linked to telomere maintenance and to antisense RNA-mediated regulatory pathways of gene expression. These results suggest widespread and distinct roles of different TRAMP complexes in the regulation of gene expression.

## Results

### Trf4p and Trf5p Modulate the Expression of Different Sets of Genes

**Figure 1:**
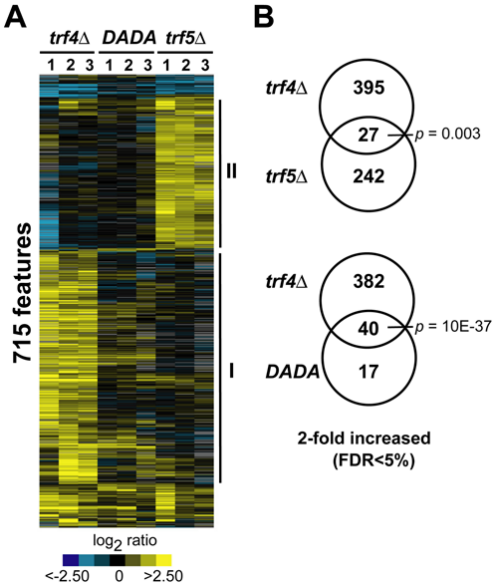
Features with altered levels in *trf4Δ*, *trf5Δ*, and polyadenylation-defective *trf4Δ*/*TRF4-DADA* mutants. (A) Hierarchical cluster of 715 features that changed at least 2-fold (FDRs<5%) in *trf4Δ*, *trf5Δ*, or *trf4Δ*/*TRF4-DADA* mutant cells (Table S1). Columns (1, 2, 3) correspond to individual experiments, rows to genes. The RNA levels in the *trf4Δ* or *trf5Δ* mutant strain relative to the WT (BY4741) strain, and in the *trf4Δ/TRF4-DADA* (*DADA*) mutant strain relative to BY4741/pNOPPATA1L strain, are colored in the yellow-blue ratio scale as indicated at the bottom. Yellow shades indicate higher transcript abundance in mutants compared to WT, and blue shades represent lower transcript abundance. Grey represents missing values. Solid lines (I and II) mark the 422 and the 269 features that were selectively increased in the *trf4Δ* (I) or the *trf5Δ* (II) mutants, respectively. (B) Venn diagrams representing overlap of transcripts that were at least 2-fold increased in mutants compared to WT. *P*-values relate to the significance of the overlap (Fisher's Exact test, right tail).

TRAMP complexes promote the exosome-assisted degradation of diverse ncRNAs and aberrant or nonfunctional RNAs [4]–[7], [14]–[16]. To identify additional specific RNA targets for the TRAMP4 and TRAMP5 complexes, we measured the relative changes of gene expression of *S. cerevisiae* cells lacking either *trf4* (*trf4Δ*) or *trf5* (*trf5Δ*) compared to wild-type (WT) cells using yeast oligo microarrays that contained features representing all annotated yeast ORFs, ncRNAs, introns, rRNA precursors, as well as some intergenic regions (IGRs) and tiled regions downstream of a few genes (see Materials and Methods). To this purpose, total RNA isolated from exponentially growing cells was reverse transcribed with a mixture of random nonamers and oligo(dT) primers. Cy5 fluorescently labeled cDNAs derived from total RNA isolated from either the *trf4Δ* or the *trf5Δ* mutants were then competitively hybridized with Cy3 labeled cDNAs from WT cells. To define a list of arrayed features determining transcripts that significantly changed expression in the *trf4Δ*, the *trf5Δ* and the *trf4Δ/TRF4-DADA* mutants (which are explained below), we arbitrarily selected those features that changed relative expression at least 2-fold (average of three biological replicates) with false discovery rates (FDRs) of less than 5% [33] (Figure 1A; a list of the selected features is provided in Table S1). Similar results were obtained by statistical analysis with Cyber-T [34] followed by selection of those features with a *p*-value of less than 0.05 [34] (for a comparison of FDRs and *p*-values see Table S2). To further visualize the relation among the 715 features selected by this analysis, we hierarchically clustered the features and experiments (Figure 1A). To identify common themes among the differentially expressed mRNAs in the *trf4Δ* and *trf5Δ* mutants, we searched for common Gene Ontology (GO) annotations among the 550 transcripts for which GO annotations were available at the *Saccharomyces* Genome Database (SGD; Table S3).

Not surprisingly, most of the 715 selected features showed increased steady-state levels in *trf* mutants (691 features, 97%), which is in agreement with the idea that Trf4p and Trf5p promote RNA degradation and their depletion hence leads to the accumulation of RNAs that are normally targeted by these proteins (Figure 1A). More interestingly, although deletions of *trf4* and *trf5* are synthetically lethal, which may suggest common functions and targets, we found that the vast majority of the affected transcripts overlapped only marginally though significantly (Figure 1B). Only 33 transcripts changed expression in both the *trf4Δ* and the *trf5Δ* mutants; among these, 27 transcripts were selectively increased (Figure 1B) and represented rRNA processing intermediates, mRNAs encoding chaperones (*SSB1*, *HSP150*, and *TIR2*) or enzymes involved in glucose metabolism (*TDH2*, *TDH3*, *ADH2*, and *PDC1*). These results suggest that *trf4* and *trf5* specifically affect very different groups of transcripts *in vivo*. However, we wish to point out that the comparison of gene expression profiles of single *trf4* and *trf5* deletion mutants may not reveal the full spectra of the *in vivo* targets; particularly in cases where either functional Trf4 or Trf5 proteins can fully substitute the absence of the other paralog.

Consistent with previous reports [6], [7], [14]–[16], 72 of the 422 features (17%) that accumulated at least 2-fold in the *trf4Δ* mutant were ncRNAs, such as snoRNAs (27 features) and RNAs derived from intergenic regions (IGRs; 20 features), or autonomously replicating sequences (ARSs; 4 features; Figure S1A, Figure S2, and Table S1). Interestingly, 13 of the 20 IGRs overlap with CUTs that have been recently mapped by massive sequencing of RNAs bound to the nuclear cap-binding protein Cbp20p isolated from conditional *trf4Δ rrp6Δ* double mutants [18], or that were accumulated in *rrp6Δ* mutants and identified with tiling arrays [19]. (A comparison of selected IGRs and CUTs is provided in a separate worksheet of Table S1.)

Ty1 retrotransposons represented the second most abundant class of transcripts with altered expression in the *trf4Δ* strain (Figure S1), as 68 out of the 98 probes specific for *TyA* and *TyB* exhibited an average 4-fold increase in relative expression levels compared to the WT strain (Figure S3A). This result was further confirmed by quantitative real-time PCR (qRT-PCR) analysis with primers specific for a sequence overlapping the *TyA* and *TyB* boundary (Figure S3B). In contrast to *trf4Δ cells*, the expression of the Ty1 elements was slightly decreased in the *trf5Δ* mutant (∼−1.5-fold) and unchanged in cells lacking the exosome subunit Rrp6p (*rrp6Δ*) (Figure S3B). Ty1 transcription and transposition is regulated by the *trans* acting antisense regulatory RTL-RNA that is transcribed divergently to *TyA* from an internal promoter and is degraded by the 5′ to 3′ exoribonuclease Xrn1p [27]. In agreement with a previous report [27], we found that the steady state levels of RTL-RNA were unaffected in *rrp6* mutants. Conversely, RTL-RNA levels were slightly increased (>1.5-fold) in the *trf4Δ* mutant and decreased (∼1.5-fold) in the *trf5Δ* mutant (Figure S3B). Since both the Ty1 elements and the negative regulator RTL-RNA were simultaneously increased in the *trf4Δ* mutant and decreased in the *trf5Δ* mutant, but remained unchanged in the exosome mutant *rrp6Δ*, we speculate that the TRAMP/exosome pathway is not involved in either the degradation of the *TyA* and *TyB* mRNAs or of the antisense regulatory RTL-RNA. However, the opposite effect of *trf4* or *trf5* deletions on the levels of the TY1- and RTL-RNAs suggests that TRAMP4 and TRAMP5 likely act through a yet uncharacterized mechanism to regulate the expression of the *TY1* locus.

Deletion of *TRF5* caused the accumulation of only 11 ncRNAs (4%) out of the 269 features (representing 220 GO annotated genes) for which we measured significantly altered expression levels (Figure S1B). This includes one snoRNA (*SNR68*), and four IGRs likely representing two CUTs (CUT857, CUT195) and the stable untranslated transcript SUT180 [19] (Table S1). However, the majority (94%) of the *trf5*-affected transcripts can be assigned to protein-coding mRNAs. A GO analysis among these messages revealed overrepresentation of mRNAs coding for cytoplasmic proteins involved in translation (e.g. 19 ribosomal protein genes, *p*<0.01) or act in diverse metabolic processes such as glycolysis (7 genes, *p*<0.01), sulphate assimilation (5 genes, *p*<0.003), and nitrogen metabolism (21 genes, *p*<0.003; GO analysis of annotated transcripts is shown in Table S3). Except for 7 messages coding for proteins acting in glycolysis (*p*<0.01), these themes could not be seen among the mRNAs that were affected in the *trf4Δ* mutant which preferentially encode nuclear proteins (116 genes, *p*<10^−17^). In conclusion, it appears that deletion of *trf4* or *trf5* affected the steady-state level of a large variety of mRNAs that function in diverse cellular pathways and may reflect in part the pleiotropic defects of *trf4Δ* and *trf5Δ* mutants [22], [24]–[26]. Our results also indicate that degradation of ncRNAs is mainly promoted by Trf4p.

### TRF4-Mediated Polyadenylation Is Dispensable for Most Targets in *Saccharomyces cerevisiae*

We next aimed at identifying the set of transcripts that require the polyadenylation activity of Trf4 for efficient degradation. For this purpose we constructed a *trf4Δ/TRF4-DADA* mutant strain, where the polyadenylation defective allele *TRF4-DADA* [5] is episomally expressed in *trf4Δ* cells under the control of the *NOP1* promoter (see Materials and Methods). Trf4p-DADA contains two aspartate to alanine mutations in the poly(A) polymerase catalytic site which renders the enzyme inactive [5]. Similar to the microarray experiments with the *trf4Δ* and the *trf5Δ* mutants, we compared transcript levels of the *trf4Δ/TRF4-DADA* mutant to that of the WT strain harboring the empty vector (BY4741/pNOPPATA1L).

Surprisingly, the expression levels of more than 90% of transcripts that were significantly altered in *trf4Δ* mutant cells were almost fully restored to WT levels by the overexpression of the *TRF4-DADA* allele (Figure 1, Figure S1C). Only 57 transcripts were more than 2-fold enriched (FDR<5%) in cells expressing the *TRF4-DADA* allele as compared to WT (Figure 1B), 18 of them (32%) are ncRNAs (Figure S1C). Although the relative abundance of most SGD annotated rRNA intermediates, snoRNAs, and IGRs was reduced in *trf4Δ/TRF4-DADA* compared to the *trf4Δ* mutant cells (Table S1), 31 ncRNAs (7 rRNA intermediates, 15 SNRs, and 9 IGRs) still exhibited increased steady-state levels (on average between 1.5-fold and 4-fold; FDR<5%) relative to the WT cells (Figure S2, Table S1). Similar results were found for the RNA component *NME1* of RNase MRP and for a group of short dubious ORFs (*YJL047C-A*, *YBR072C-A*, *YGR121W-A*, and *YBR182C-A*) that were also enriched in *rrp6Δ* mutants [19] and, hence, most likely do not encode proteins but rather correspond to CUTs. Some of these ncRNAs are highly abundant such as rRNAs and snoRNAs. It therefore appears that highly expressed and structured RNAs strongly depend on the polyadenylation activity of Trf4p although they represent only a minor fraction (10%) of Trf4 targets. However, this fraction may recruit a considerable amount of Trf4 complexes *in vivo* and thus, a substantial fraction of the total RNA turnover mediated by Trf4p may depend upon Trf4 catalytic activity.

### Trf4p Stimulates the Degradation of a Subgroup of Introns

**Figure 2:**
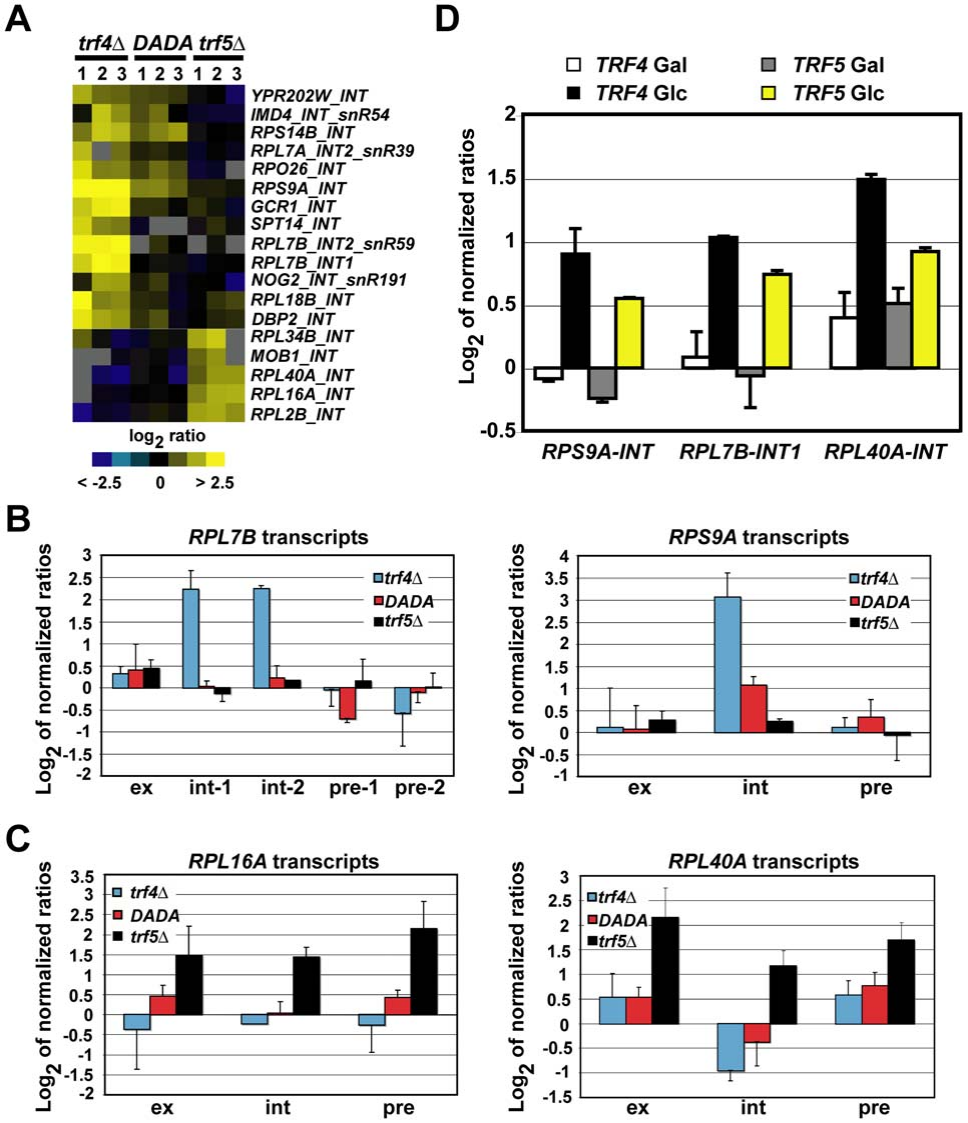
Trf4p promotes polyadenylation-independent degradation of introns. (A) Microarray data for 18 introns that changed more than 2-fold (FDRs<5%) in *trf4Δ* or *trf5Δ* mutants. Overexpression of Trf4p-DADA in *trf4Δ* mutant cells reduced steady-state levels of most of these introns to WT levels. Microarrays are the same as shown in Figure 1. Introns containing snoRNAs (*snR*) are indicated. (B) Relative changes of the expression of *RPL7B* (left panel) and *RPS9A* (right panel) introns, pre-mRNAs, and mature mRNAs as revealed by exon (ex), intron (int), and intron-exon junction probes (pre) present on the microarrays. Int-1 and int-2 refer to the first and the second intron of the *RPL7B* gene; pre-1 and pre-2 refer to the respective intron-exon junction probes of the *RPL7B* pre-mRNA. The height of the bar represents average log_2_ ratios from triplicate microarray data (Dataset S1); error bars show the standard deviation. (C) Relative changes of expression of *RPL16A* (left panel) and *RPL40A* (right panel) introns (int), pre-mRNAs (pre) and mature mRNAs (ex). Data was extracted from triplicate microarray data as described above. (D) Depletion of Trf4p or Trf5p promotes intron stability *in vivo*. Total RNA was purified from *trf4Δ trf5Δ* mutant strains either expressing *TRF4* or *TRF5*, which were transcribed under the *pGAL1* promoter. Cells were initially grown in a galactose containing medium (*TRF4* Gal; *TRF5* Gal) and then shifted to a glucose containing medium (YPD) at 30°C (*TRF4* Glc; *TRF5* Glc) for 1 h. Accumulation of introns of *RPS9A* and *RPL40A* and of the first intron of *RPL7B* was determined by qRT-PCR with intron-specific primers. The steady-state levels of introns in each sample was calculated as log_2_ of normalized ratios relative to the *t_0_* time point, which corresponds to the cultures immediately before the galactose to glucose shift (for details see Materials and Methods). The values represent averages from two independent qRT–PCR analyses.

In addition to ncRNAs such as rRNA intermediates, snoRNAs, and IGR RNAs/CUTs, our microarray data showed that a group of introns, some of which containing snoRNAs, were specifically accumulated (2- to 8-fold; FDR<5%) in either the *trf4Δ* mutant (Trf4-dependent introns) or the *trf5Δ* mutant (Trf5-dependent introns; Figure 2A, Table S1). To rule out that accumulation of these introns simply reflects increased transcript levels of the pre-mRNAs, we compared the relative changes of intron abundance with that of the corresponding pre-mRNAs and mature mRNAs as revealed with arrayed probes that specifically detect intron-exon junctions and exons (Figure 2B and 2C). We found that unlike introns, the corresponding pre-mRNAs and mature mRNAs were not significantly changed in the *trf4Δ* mutant compared to WT cells. Likewise, the cognate pre-mRNAs and mature mRNAs of the Trf4-dependent introns were also unchanged in the *trf5Δ* and *trf4Δ/TRF4-DADA* mutants (Figure 2B, Table S1). Thus, this analysis indicates that only spliced-out introns, such as those of the *RPS9A*, *RPL7B*, and *GCR1* genes, specifically accumulate in the *trf4Δ* mutant. To validate this finding, we carried out qRT-PCR experiments with primers either specific for the introns, the pre-mRNAs, or the mRNAs of *RPS9A*, *RPL7B*, and *GCR1*. Consistent with our microarray data, the levels of introns but not those of pre-mRNAs or mature mRNAs were increased in the *trf4Δ* mutant compared to WT cells (Figure S4 and results not shown).

As Trf4p promotes the exosome-mediated degradation of targeted RNAs through its polyadenylation activity [1], [5]–[7], we also analyzed the steady-state levels of introns in the *rrp6Δ* exosome mutant and in *trf4Δ/TRF4-DADA* mutant cells. Similarly to what has been observed in the *trf4Δ* mutant, introns accumulated in cells lacking *rrp6* but no significant change in the expression of the pre-mRNAs and mature mRNAs of *RPS9A*, *RPL7B*, and *GCR1* was detected (Figure S4 and data not shown). Conversely, overexpression of the *TRF4-DADA* allele in *trf4Δ* cells restored WT levels for nine of the 13 Trf4-dependent introns, or reduced their abundance to values slightly above (1.5 fold) the WT levels (Figure 2A, Figure S4, Table S1, and results not shown). This indicates that the polyadenylation-defective Trf4-DADA protein also participates in the regulation of “normal” steady-state levels of introns *in vivo*.

Unlike to what we observed in the *trf4Δ* mutant, however, the increased levels of the Trf5-dependent introns (Figure 2A; *RPL16A-INT* and *RPL40A-INT* in Figure S4) coincided with similar amounts of the related pre-mRNAs and mature mRNA transcripts (Figure 2C, Table S1, and data not shown), strongly suggesting that accumulation of introns in the *trf5Δ* mutant reflects increased relative abundance of unspliced pre-mRNAs.

To test whether Trf5p could efficiently target spliced-out introns in the absence of Trf4p, we analyzed intron accumulation by qRT-PCR experiments upon conditional *TRF4* or *TRF5* depletion. In this experiment, total RNA was isolated from a *trf4Δ trf5Δ* double mutant strain complemented with a plasmid either expressing *TRF4* or *TRF5* under the control of the *GAL1* promoter (for details see Materials and Methods). As shown in Figure 2D, an one hour shift of cells to media supplemented with glucose to repress expression of *TRF4* or *TRF5*, led to an enrichment of all the introns tested. Conversely, no change in the abundance of introns was observed in control experiments performed with total RNA purified from WT cells that were transformed with the empty vector (BY4741/pYC6-CT; results not shown).

Taken together, these results suggest that Trf4p likely promotes the exosome-mediated degradation of a group of spliced-out introns through a mechanism that is not dependent on polyadenylation. In addition, as depletion of *TRF5* in *trf4Δ* cells caused intron accumulation *in vivo*, we infer that Trf4p and Trf5p are functionally redundant for intron decay (Figure 2D).

### TRAMP4 Interacts with Introns *In Vivo*

**Figure 3:**
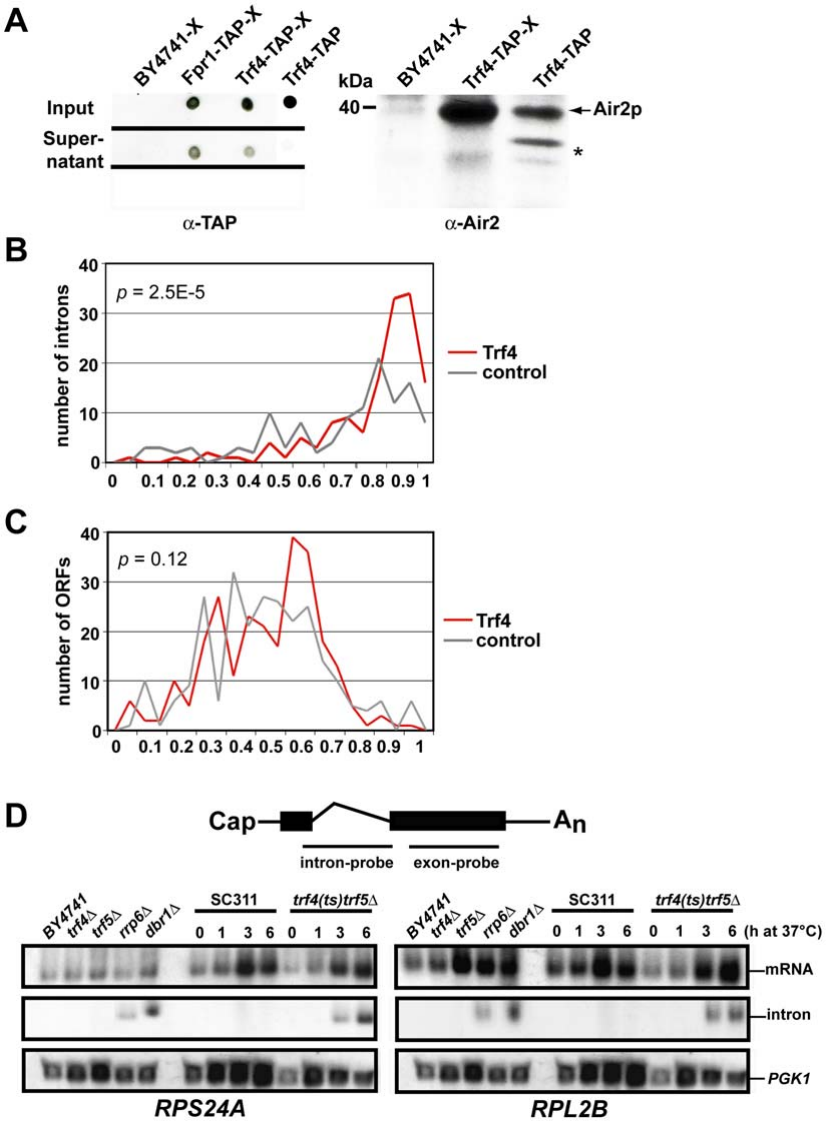
Selective enrichment of introns in TRAMP4 affinity–isolates. (A) RNA–protein complexes were purified from extracts of cells expressing recombinant Trf4-TAP protein after *in vivo* cross-linking with formaldehyde (Trf4-TAP-X). Extracts of BY4741 cells and of cells expressing Fpr1-TAP were used as control (BY4741-X; Fpr1-TAP-X). Affinity purification of the Trf4-TAP protein complex or Fpr1-TAP from the WCEs was monitored by dot blot (left panel) and immunoblot (right panel) analyses with antibodies detecting the calmodulin-binding region of the TAP-tag (α-TAP) or the Air2p subunit of the TRAMP4 complex (α-Air2). Input and supernatant correspond to cross-linked WCEs before and after immunopurification, respectively. Affinity purified TAP-tagged Trf4 protein complex from non cross-linked WCEs was used as a positive control for the purification procedure (Trf4-TAP). A molecular weight marker is shown next to the immunoblot; * denotes likely Air2p degradation products. (B, C) Percentile rank analysis of immunopurified RNA preparations. RNAs enriched by TAP-tag affinity purification was comparatively analyzed to total RNA purified from WCEs with DNA microarrays. The enrichment profiles of introns (B) and the respective ORFs (C) were created by binning average percentile ranks from three biological replicates of Trf4-TAP or control IPs (two replicates of Fpr1-TAP and one untagged cells) into 0.05 unit bins. *P*-values to estimate the difference between Trf4 and control data were calculated with the Kolmogorov-Smirnov test. (D) Northern analysis with intron- and exon- specific probes in RNA surveillance mutants (*trf4Δ*, *trf4Δ/TRF4-DADA*, *trf5Δ*, and *rrp6Δ*) and in the debranching enzyme *dbr1Δ* mutant strain. Strains were grown at 30°C in YPD, except *SC311* and *trf4(ts)trf5Δ* [25] that were shifted to 37°C for the indicated times prior to RNA extraction.

To identify RNAs associated with TRAMP4, we performed *in vivo* cross-linking and ribonucleoprotein-immunopurification experiments followed by microarray analysis of bound RNAs (X-RIP-Chip). Cells expressing recombinant tandem-affinity purification (TAP)-tagged Trf4 protein were cross-linked with formaldehyde, and Trf4-containing ribonucleoprotein complexes were recovered by affinity selection on IgG-coupled beads (see Materials and Methods). Cells expressing TAP-tagged Trf4 proteins fully restore Trf4 functions and were previously used to purify functional TRAMP complexes [5]. As a control for non-specifically enriched RNAs, the same experiment was done with untagged WT cells and with cells expressing Fpr1-TAP, a peptidyl-prolyl-cis-trans-isomerase not expected to bind RNA. About 70% of Trf4-TAP and 60% of Fpr1-TAP was captured from the whole cell extract (WCE) as shown by dot-blot analysis (Figure 3A, left panel). Moreover, Air2p, a well-known component of the TRAMP4 complex [5],[6], co-purified with crosslinked Trf4-TAP but was absent in control purifications performed with untagged WT cells (Figure 3A, right panel).

We isolated total RNA from extracts (input) and from the immunopurified samples and labeled cDNAs derived from the RNAs with Cy3 and Cy5 fluorescent dyes, respectively. The differentially labeled samples were mixed and competitively hybridized on yeast oligo arrays. In this assay, the ratio of the two RNA populations at a given array element provides a measurement for enrichment of the respective RNA with the TRAMP4 complex [35],[36]. Because of the relatively high variation of array data between biological replicates, we rank ordered the data and determined percentile ranks for each analyzed feature (0, no enrichment; 1 high enrichment; Dataset S2).

In agreement with known functions of TRAMP4 on ncRNAs, we found that many small and stable ncRNAs such as snoRNAs and tRNAs were highly enriched in purified cross-linked TRAMP4 complexes. However, these transcripts were also strongly enriched in the control isolates and thus, only limited conclusions can be drawn from this analysis. Nevertheless, despite the high background from small ncRNAs in these experiments, we found that spliced-out introns were selectively enriched in the Trf4-TAP RNA isolates when compared to control isolates (Kolmogorov-Smirnov test: *p* = 2.5×10^−5^; Figure 3B, data for all intron probes are shown in Table S4). The corresponding probes for the mature mRNAs were not enriched (*p* = 0.12; Figure 3C), suggesting specific association of the TRAMP4 complex with spliced introns but not with the respective pre-mRNAs. This finding is consistent with our previous observation for accumulation of certain introns but not of the corresponding mRNAs in the *trf4Δ* mutant (Figure 2 and Figure S4): For 11 (78%) of the 14 introns that were significantly changed in either *trf4Δ* and *trf5Δ* mutants (Figure 2A) and for which X-RIP-Chip data were available, we found higher ranking with TRAMP4 complexes compared to controls (Table S4), whereas no such preference was seen for the respective ORF probes (two of the six corresponding ORFs were higher ranked with TRAMP4 complexes).

We further analyzed whether expression levels of the TRAMP4 associated introns were commonly changed in single *trf4Δ* or *trf5Δ* mutants. The relative expression levels for 48 introns that were preferentially enriched with TRAMP4 complexes (percentile ranks greater than 0.85) compared to the controls, were mostly unchanged in either *trf4Δ* or *trf5Δ* mutants (average log_2_ ratios = 0.035 and −0.1 in *trf4Δ* and *trf5Δ*, respectively; Table S4). Possibly, Trf4p and Trf5p act redundantly in the decay of most spliced-out introns and therefore no changes of relative expression levels can be seen in single mutants. To further corroborate this idea, we measured the expression of introns for the two genes *RPS24A* and *RPL2B* that were enriched in TRAMP4 affinity isolates (average percentile ranks of 0.83 and 0.9, respectively) but for which expression levels were not significantly changed in neither *trf4* nor *trf5* mutants, by Northern blot analysis with intron specific probes. As expected, these introns were detectable in total RNA derived from the *rrp6Δ* exosome mutant and from the mutant of the debranching enzyme *dbr1*Δ, which was used as a positive control for intron detection [37]–[39] (Figure 3D). However, both introns could not be readily detected in total RNA derived from either the *trf4Δ* or the *trf5Δ* single mutant strains, but they were strongly accumulated in RNA samples isolated from conditional *trf4(ts)trf5Δ* double mutants. This result is reminiscent of our finding for the *RPS9A*, *RPL7B*, and *RPL40A* introns that became increasingly enriched in conditional double mutants (Figure 2D). In conclusion, these data strongly suggest functional redundancy between Trf4p and Trf5p in the degradation of introns *in vivo*.

### The *trf4Δ* Mutant Is Defective in the Turnover of Subtelomeric RNAs

**Figure 4:**
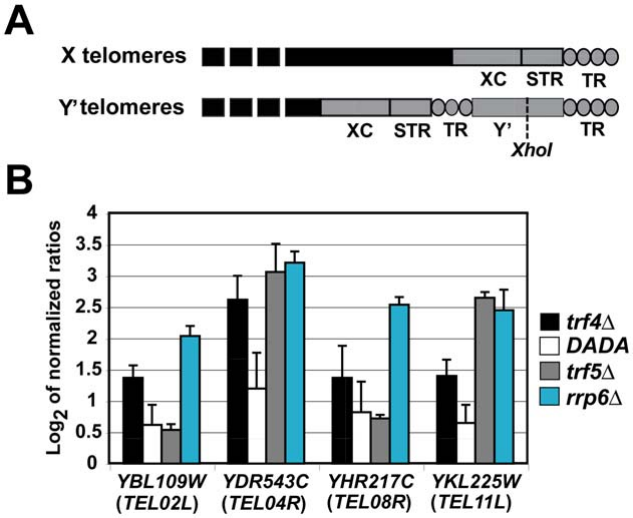
Subtelomeric transcripts are detected in strains defective in RNA surveillance pathways. (A) Schematic representation of yeast telomeres. X-type telomeres consist of the X element core sequences (XC), the subtelomeric repeats (STR), and the telomeric repeats at the ends of the chromosomes. The Y′-type telomeres contain a subtelomeric Y′ DNA sequence located within the telomeric repeats that is not found in X telomers. A conserved *Xho*I restriction site specific for Y′ sequences is shown. (B) Bar diagram representing the qRT–PCR analysis for four subtelomeric ORFs transcribed from X- and Y′-type telomeric regions. Corresponding telomeres are indicated in brackets. RNA amounts were normalized to *ACT1* mRNA and are compared relative to the isogenic WT strain. Relative changes of transcript abundances (log_2_ ratio scale) represent averages from two independent qRT-PCR analyses. The same RNA preparation was used for the microarray analysis shown in Figure 1A.

Yeast telomeres (TEL) consist of a complex mosaic of telomeric and subtelomeric sequences, where the X element sequence is the only region common to all chromosome ends. Some subtelomeric regions contain a conserved helicase-encoding repetitive sequence (Y′ sequence) located within terminal telomeric repeats (TR; Figure 4A) [40]. It has recently been reported that cryptic transcripts originating from transcriptionally repressed loci, such as *TEL05L*, accumulate in strains lacking components of the TRAMP4 or the exosome complex [30]. Consistent with this, we found that transcripts spanning across *HMLα1* and *ARS318-HMR* of the silenced mating cassettes and across putative subtelomeric ORFs (*YBL109W*, *YDR543C*, *YHR217C*, and *YKL225W*) were highly abundant in the *trf4Δ* and the *rrp6Δ* mutants (Table S1, Figure 4B, and Figure S5A). In particular, the subtelomeric transcripts originate from telomeres that either contain (*TEL02L*, *TEL04R*, and *TEL08R*) or lack (*TEL11L*) the Y′ sequence element (Figure 4B). Similar to what has been reported previously [30], they are commonly oriented in the 5′ to 3′ direction towards the centromere (results not shown). Neither subtelomeric RNAs transcribed from the opposite strand towards the telomeres nor telomeric TERRA RNAs [41] were detected (results not shown). Although overexpression of *TRF4-DADA* reduced the abundance of these subtelomeric RNAs in *trf4Δ* mutant cells, their steady-state levels were still about 1.5–2.2 fold higher than in WT cells (Figure 4B). This decrease in the abundance of subtelomeric RNAs seen in the *trf4Δ/TRF4-DADA* mutant indicates that the polyadenylation activity of Trf4p may enhance the degradation of these RNA molecules *in vivo* (Figure 4B). Increased expression for two of these RNAs (*YDR543C* and *YKL225W*) was also found in strains deficient of *trf5* as shown by qRT-PCR experiments (Figure 4B). Interestingly and in contrast to *trf4Δ* mutants, the *trf5Δ* mutant exhibited also changes in the relative expression of factors that positively (*SIR2*, *SIR3*, and *MCM10*) or negatively (*SAS5*) regulate chromatin silencing (Figure S5B). Thus, it could be that accumulation of subtelomeric RNAs in the *trf5Δ* mutant reflects defects in pathways other than RNA turnover.

### Telomere Shortening Correlates with Increased *TLC1* Expression in *trf4Δ* Mutants

**Figure 5:**
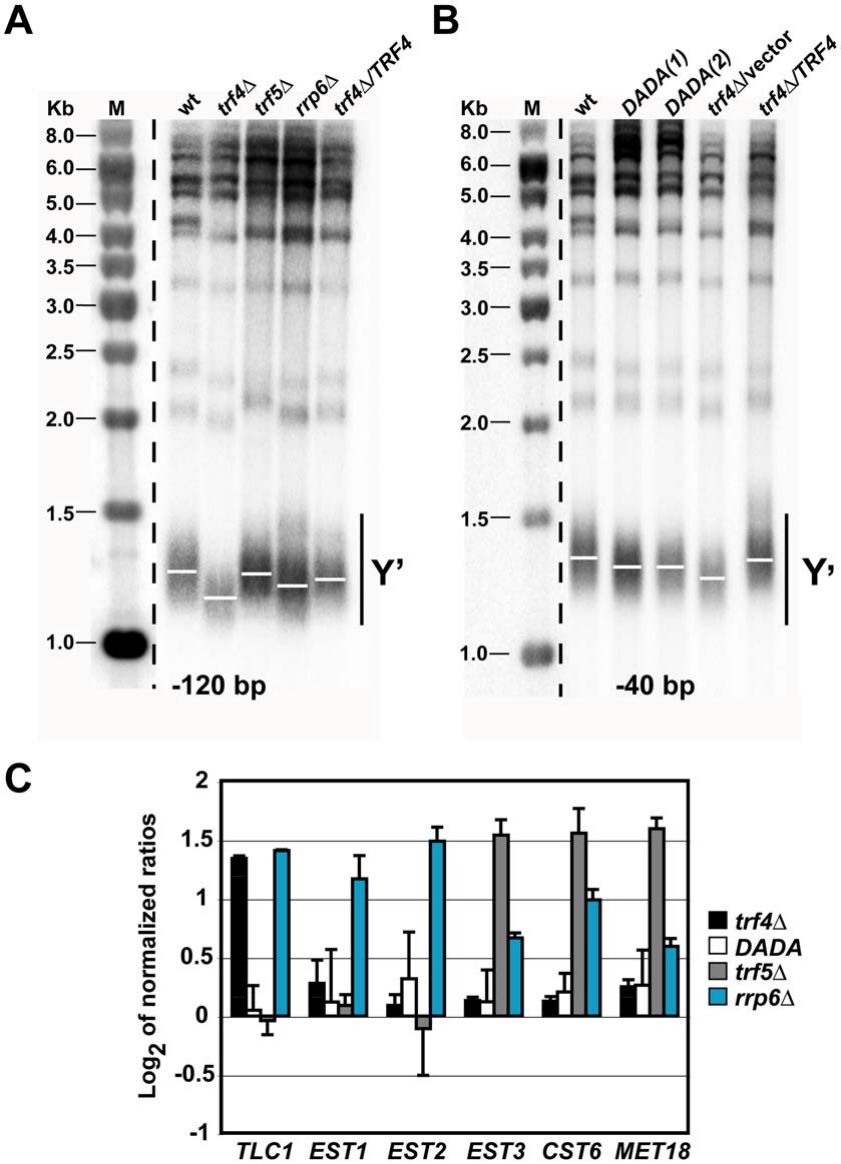
Trf4p promotes telomere maintenance and *TLC1* regulation through a polyadenylation-independent mechanism. (A, B) Southern blot analyses of Y′-type telomeres was carried out with *Xho*I-digested DNA of RNA surveillance mutants (*trf4Δ*, *trf4Δ/TRF4-DADA*, *trf5Δ*, and *rrp6Δ*) of *S. cerevisiae* probed with telomeric sequences. Mutant strains are indicated at the top: wt corresponds to the wild-type BY4741 strain; *trf4Δ*/vector represents the *trf4Δ* mutant strain transformed with pNOPPATA1L. *DADA*(1) and *DADA* (2) represent two different clones of the *trf4Δ/TRF4-DADA* mutant. A marker (M) for DNA sizes in kilobases is shown to the left. Solid black bars flanked by Y′ shows the *Xho*I-digested Y′ DNA fragments, where white bars refer to the position of average size fragments. Numbers in brackets refer to the average shortening of the Y′-containing telomeres in the *trf4Δ* and *trf4Δ*/*TRF4-DADA* mutants relative to wt. (C) qRT–PCR analysis examining the levels of specified mRNAs and ncRNAs (*TLC1*) in four RNA surveillance mutants (*trf4Δ*, *trf4Δ/TRF4-DADA*, *trf5Δ*, and *rrp6Δ*). RNA amounts were normalized to *ACT1* mRNA and compared to the isogenic WT strain. Relative changes of transcript abundances (log_2_ ratio scale) represent averages from two independent qRT-PCR analyses. The same RNA was used for the microarray analysis presented in Figure 1A.

To test whether any correlation existed between subtelomeric RNA accumulation and the structural integrity of telomeres, we performed Southern blot experiments with *Xho*I digested genomic DNA to determine the length of Y′ containing telomeres (Figure 5A and 5B). Y′ telomeres were on average shortened by ∼120 bp in the *trf4Δ* mutant and ∼40 bp in the *rrp6Δ* exosome mutant (Figure 5A). Conversely, the length of Y′ telomeres was similar to that of the WT strain in the *trf5Δ* mutant or in *trf4Δ* mutant cells complemented with a plasmid (pNOPPATA1L) carrying the WT allele of *TRF4* (Figure 5A). Intriguingly, shortening of telomeres in the *trf4Δ* mutant was also strongly suppressed by overexpression of *TRF4-DADA*, where telomeres were about 40 bp shorter than in the WT strain (Figure 5B). These results showed that telomere shortening in *trf4Δ* cells is a reversible event achieved by reintroduction of episomally expressed Trf4 proteins. In addition, our results strongly suggest that Trf4p exerts a role in telomere maintenance mainly through a mechanism that is independent of polyadenylation.

The results from these Southern blotting experiments do not indicate any straight correlation between telomere shortening and accumulation of the subtelomeric RNA molecules. In fact, although subtelomeric RNAs were more abundant in the *rrp6Δ* mutant compared to the *trf4Δ* or the *trf4Δ/TRF4-DADA* mutant strain (Figure 4B), only disruption of *trf4* resulted in a severe shortening of the telomeres. Moreover, some subtelomeric RNAs (*YDR543C* and *YKL225W*; Figure 4B) also highly accumulated in the *trf5Δ* mutant, which did not show any recognizable change in telomere length (Figure 5A).

To investigate whether misregulation of the telomerase components could be the cause of the telomere shortening, we carried out qRT-PCR experiments with primers specific for the *TLC1*, *EST1*, *EST2*, and *EST3* subunits (Figure 5C) [42],[43]. In the *rrp6Δ* mutant, the steady-state levels for the mRNAs encoding these telomerase subunits were more than 2-fold increased compared to the WT strain, suggesting a consequent increase in the activity of the holoenzyme in this mutant. In contrast, only the *TLC1* RNA was 2.6-fold increased in the *trf4Δ* mutant, whereas no change was detected for *EST1*, *EST2*, and *EST3* mRNAs levels (Figure 5C). It was reported that overexpression of *TLC1* causes telomere shortening in yeast because of the specific sequestration of the telomeric factors yKu70 and yKu80, which promote telomerase recruitment [42]. Thus, the imbalance in the expression level between *TLC1* and the other subunits of the telomerase may in part account for for the telomere shortening observed in the *trf4Δ* mutant. This hypothesis is further supported by the observation that overexpression of the *TRF4-DADA* allele not only suppressed the telomeric defect of the *trf4Δ* mutant, but also coincided with the restoration of expression of the *TLC1* RNA subunit to WT levels (Figure 5C). Furthermore, these results indicate that Trf4p promotes *TLC1* turnover through a polyadenylation-independent mechanism. Consistent with the proposed connection between *TLC1* overexpression and telomere shortening, we found no change of *TLC1* RNA abundance in the *trf5Δ* mutant (Figure 5C). It is noteworthy, however, that the unaltered telomere length found in the *trf5Δ* mutant might also reflect increased expression levels (>3-fold) of factors such as *EST3*, *CST6*, and *MET18*, which participate in the maintenance of telomeres *in vivo* (Figure 5C) [44].

### Trf4p and the Exosome Promote Degradation of Antisense RNAs

**Figure 6:**
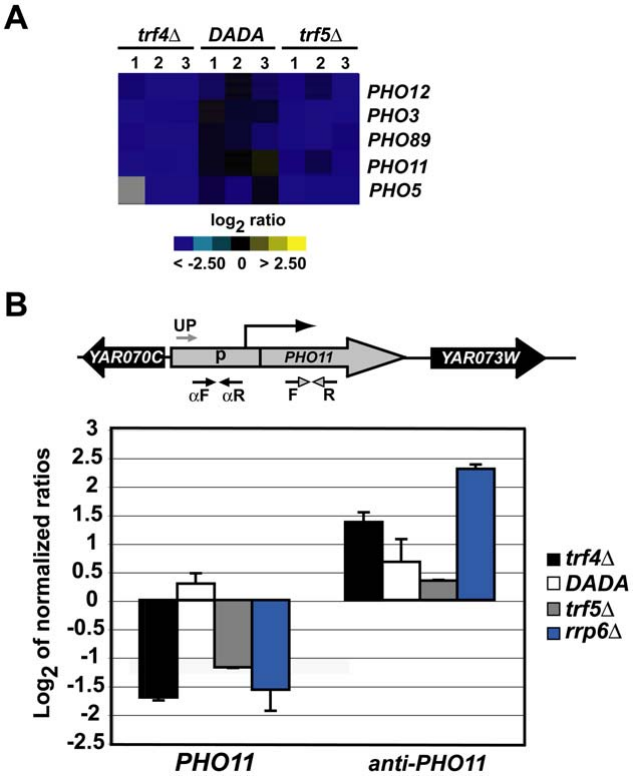
Trf4p participates in antisense RNA–mediated regulation of genes of the phosphate metabolism. (A) Relative expression levels of *PHO3*, *PHO5*, *PHO11*, *PHO12*, and *PHO89* in the *trf4Δ*, *trf5Δ* and *trf4Δ/TRF4-DADA* (DADA) mutants. Microarrays are the same as those shown in Figure 1A. (B) Strand specific qRT-PCR analysis examining relative changes of expression of *PHO11* and *anti-PHO11* in RNA surveillance mutants (*trf4Δ*, *trf4Δ/TRF4-DADA*, *trf5Δ*, and *rrp6Δ*). Schema above the bar diagram represents the *PHO11* gene locus: The grey arrow (UP) indicates the position of the UP-αPHO11 primer used for strand specific synthesis of *anti-PHO11* cDNA; convergent solid arrows (αF and αR) indicate the primer pairs anti-pho11-Fw and anti-pho11-Rv used for the quantification of the *anti-PHO11* cDNA; and convergent grey arrowheads (F and R) show the location of pho11-Fw and pho11-Rv primers used for the quantification of the *PHO11* cDNA. Data was normalized to *ACT1* mRNA. Bars indicate relative changes of transcript levels (log_2_ normalized ratios) in mutants compared to WT strains and are averages from two independent analyses. The same RNA was used for the microarray analysis shown in Figure 1.

Besides the many mRNAs for which relative expression levels were significantly increased in strains devoid of Trf4p or Trf5p, the expression of a few genes including those coding for proteins involved in phosphate metabolism (*PHO3*, *PHO5*, *PHO11*, *PHO12*, and *PHO89*) were significantly decreased (Table S1). The relative abundance of these mRNAs was almost fully restored to WT levels by the overexpression of the *TRF4-DADA* allele as shown by microarray and qRT-PCR experiments (Figure 6, Figure S6, and Table S1).

Such reduced expression of the *PHO* genes has also previously been observed in nuclear exosome mutants [16]. Particularly for *PHO84* and *PHO5*, it was shown that Rrp6p affects the stability of corresponding antisense RNAs involved in the transcriptional control of their cognate sense mRNAs [19],[29],[45].

To assess whether the decreased levels of *PHO5*, *PHO11*, and *PHO89* mRNAs in *trf4Δ*, *trf5Δ*, and *rrp6Δ* exosome mutants correlates with increased levels of corresponding antisense RNAs, we carried out strand-specific qRT-PCR experiments with primers specific for antisense RNAs that span across the *PHO* promoter regions (Figure 6B, Figure S6). Antisense RNAs could be detected in all the strains tested, including the WT strain, however, their levels were more than 2-fold increased in *trf4Δ* and *rrp6Δ* but not in *trf5Δ* mutants. The abundance of the antisense RNAs was decreased to WT levels by overexpression of *TRF4-DADA* (Figure 6B, data for *PHO5* and *PHO89* are shown in Figure S6). Therefore, similar to *PHO84* [29], the expression of *PHO5*, *PHO11*, and *PHO89* is likely modulated by antisense RNAs, the degradation of which is promoted by the exosome and the Trf4 protein, and does not require the polyadenylation activity of Trf4p. In addition, although loss of Trf5p did not cause any change in the expression of the *PHO* antisense transcripts, the reduced steady state level of the *PHO5*, *PHO11*, and *PHO89* mRNAs suggests that Trf5p, through an yet unknown mechanism, may also participate with Trf4p and the exosome in fine tuning the expression of the *PHO* genes.

## Discussion

### Identification of RNAs Regulated by Trf4p and Trf5p

Trf4p and Trf5p are non-canonical poly(A) polymerases that activate RNA turnover and quality control pathways by targeting aberrant and short-lived RNA substrates to the nuclear exosome for degradation [1], [4]–[7],[15],[20]. *Trf4* and *trf5* are synthetically lethal and depletion of Trf5p strengthens the defects in RNA maturation of *trf4Δ* mutants, suggesting that Trf4p and Trf5p have partially overlapping functions *in vivo* [6],[7],[20]. To globally investigate the extent of functional redundancy and to systematically identify Trf4p- and Trf5p-specific RNA targets, we used microarrays to compare RNA expression profiles of *S. cerevisiae* mutant strains lacking Trf4p or Trf5p with that of WT cells (Figure 1, Table S1). We found that almost all (>90%) of the 715 features that were at least 2-fold changed, were selectively increased in either the *trf4Δ* or the *trf5Δ* mutants. This finding is in agreement with known functions of these proteins in RNA degradation and their depletion is therefore expected to lead to the accumulation of RNA targets [1], [5]–[7],[20]. However, in contrast to the proposed functional redundancy of Trf4p and Trf5p, we found that *trf4* and *trf5* deletion affected barely overlapping sets of transcripts (Figure 1A). Such heterogeneity of the genes with altered expression was previously reported for different mutants of the exosome complex, possibly reflecting differential target specificities by the different subunits of the complex [16].

Interestingly, the *trf4Δ* and the *trf5Δ* mutants differed in the number of ncRNAs that accumulated in the cell. NcRNAs represented 17% and 4% of the transcripts that were selectively increased (>2-fold, FDR<5%) in *trf4Δ* and *trf5Δ* mutants, respectively. However, our microarrays cover only a fraction of the experimentally defined CUTs derived from intergenic regions (IGRs) [18],[19]. Moreover, functional antisense RNAs are also not detected with our oligo arrays including the antisense RNAs spanning the promoter region of different *PHO* genes (Figure 6, Figure S5) [29],[45]. Nevertheless, application of qRT-PCR with antisense-RNA specific primers suggests that both TRAMP4 and TRAMP5 complexes as well as the exosome participate in RNA-mediated regulatory mechanisms to modulate the expression of several *PHO* genes [29] but that only TRAMP4 triggers the exosome-mediated degradation of regulatory antisense PHO RNAs *in vivo*.

In conclusion and consistent with previous reports [6], [7], [14]–[16], our experiments support a major role for Trf4p in the exosome-mediated degradation of ncRNAs and suggest that TRAMP4 and TRAMP5 may function on specific subsets of RNAs *in vivo*. However, it remains to be further investigated how the TRAMP4 and the TRAMP5 complexes achieve specificity for their selective targets. TRAMP4 and TRAMP5 consist of structurally similar protein complexes [1],[5],[7], therefore specificity could be conferred by protein-protein interactions that are engaged by Trf4p or Trf5p and by the Air1p or Air2p subunits [5]–[8],[20]. Misfolding of the RNPs or the association of proteins with aberrant RNAs may act as selectivity factors that eventually favor the recruitment of either TRAMP4 or TRAMP5 to the RNP target.

### The Polyadenylation Activity of Trf4p Promotes Degradation of a Subset of RNAs

Several groups have previously demonstrated that the polyadenylation activity of Trf4p stimulates the exosome-mediated degradation of different RNA species *in vivo* and *in vitro* [4]–[6]. Consistently, rRNA processing intermediates, snRNAs, snoRNAs, and a few CUTs accumulate as non-polyadenylated molecules in the *trf4Δ* or the *trf5Δ* mutant strains [6],[7],[14],[15],[20]. Intriguingly, even though polyadenylation activity is required for the degradation of highly structured RNAs *in vitro*, it was reported that a polyadenylation-defective form of Trf4p (Trf4p-DADA) can also activate degradation of RNAs by the exosome [9],[30]. Moreover, a polyadenylation-defective *trf4* mutation can rescue the lethality of *trf4* and *trf5* double mutants [7]. These findings lead to a model, which proposes that the polyadenylation activity of Trf4p may not generally be necessary to guide RNA to the exosome for degradation. However, the universality of this model and whether there might be sets of RNAs that differentially depend on polyadenylation activity has not been addressed so far. Surprisingly, we found that Trf4p-DADA almost fully suppressed the altered gene expression profile of the *trf4Δ* mutant upon overexpression (Figure 1, Table S1). This finding generally supports and extends the model introduced above: Since Trf4p-DADA only partially rescues the accumulation of selected RNAs in the *trf4Δ* mutant, we suggest that the polyadenylation activity of Trf4p enhances the degradation of most target RNAs by the exosome, but this function is not essential. Polyadenylation in combination with the helicase activity of Mtr4p, which has a marked preference for binding to poly(A) RNAs [46], may be required for digestion of highly structured RNAs. This may be exemplified by the higher fraction of non-coding RNAs among the RNAs that remained accumulated in *trf4Δ* mutants overexpressing TRF4-DADA (Figure S1).

However, additional mechanisms may account for the suppression of the *trf4* mutation by Trf4p-DADA. For instance, since the TRAMP complexes share common subunits, an intriguing speculation is that Trf4p-DADA, in the context of TRAMP4, recruits Trf5p to target RNAs. Trf5p then adds poly(A) tails to facilitate exosome-mediated degradation. In agreement with this idea is the finding that deletion of *trf5* in the polyadenylation-defective *trf4-236* mutant enhanced the defect in the degradation of CUTs compared to either single mutant [6]. Although this model could explain some of the observed effects in our system (Figure 2D), it cannot account for the observation that Trf4p-DADA rescues the lethality of *trf4 trf5* double mutants [7].

### Trf4p and Trf5p Stimulate the Degradation of Introns

Whereas the mechanism of splicing has been extensively investigated, very little is known about the degradation of spliced-out introns [38],[39],[47]. In this work, we showed by combined crosslinking-RNA-immunopurification experiments that TRAMP4 likely interacts directly with introns *in vivo* (Figure 3). We also provide experimental evidence supporting a role for TRAMP4 in the degradation of spliced-out introns, which is largely independent of the polyadenylation activity of Trf4p (Figure 2, Figure S4). However, we could not find a simple correlation between the introns that were highly associated with TRAMP4, and the relative changes of expression in single *trf4Δ* or *trf5Δ* mutants. Moreover, because the expression levels of some introns became exclusively affected in *trf4 trf5* double mutants, Trf5p may promote the breakdown of introns in the absence of Trf4p suggesting functional redundancy between Trf4p and Trf5p in intron decay. Further experiments are required to unravel the contributions of different TRAMP complexes in intron decay and to delineate the exact extent of functional redundancy.

We envisage that after splicing, intron lariats are rapidly converted into linear forms by the debranching enzyme Dbr1p. Subsets of specific linear introns are then captured by TRAMP complexes to be eventually degraded by the nuclear exosome. Additional pathways may also exist, which involve the 5′ to 3′ exoribonuclease Rat1 and the endonuclease RNaseIII. In fact, lariats that contain RNaseIII binding sites can also undergo internal cleavage by RNaseIII irrespective of the Dbr1-mediated debranching, generating cleavage products that are eventually degraded by exoribonucleases [38].

### Trf4p Functions in Subtelomeric RNA Silencing and Telomere Maintenance

Transcription at heterochromatin regions was recently reported to occur in *S. cerevisiae* and *S. pombe* cells that lack Trf4p or Rrp6p [30],[48]. Consistent with these reports, we detected the accumulation of a number of RNAs originating from silent mating type cassettes and subtelomeric transcripts in the *trf4Δ* and *rrp6Δ* mutants, and to a lower extent in *trf5Δ* cells (Figure 5, Figure S5). This activity is partially dependent on the polyadenylation activity of Trf4p and on a functional exosome (Figure 4B). Although further experiments are needed to elucidate how Trf4p and the exosome contribute to the silencing of heterochromatin domains, we hypothesize that during degradation of subtelomeric RNAs, TRAMP4, and the exosome modulate the interaction or the accessibility of chromatin remodeling factors such as Sir2 and Set1 [49],[50] within sites of heterochromatin formation [49],[50]. There is a growing body of evidence that suggests interactions of Trf4p and chromatin remodeling factors (reviewed in [23]).

Transcription of heterochromatin regions can regulate important physiological pathways. In *S. cerevisiae* and *S. pombe* strains with mutations in TRAMP or exosome components, accumulation of heterochromatic CUTs has been linked to changes in rDNA copy numbers [30],[31]. Likewise, high levels of telomeric repeat-containing RNAs (TERRA) were shown to act in telomere maintenance in mammalian cells [51] and yeast [41]. In addition to alteration in the rDNA copy number [30], we discovered that the *trf4Δ* mutant of *S. cerevisiae* exhibits a severe shortening of telomeres and that telomeres were only mildly reduced in the *rrp6Δ* mutant (Figure 5A). Similarly to what was reported for the regulation of the rDNA repeats [30], telomere maintenance was not strictly dependent on the polyadenylation activity of Trf4 (Figure 5B). Although accumulation of subtelomeric RNAs may perturb the chromatin integrity at the telomeres and negatively affect the telomerase activity, additional mechanisms probably account for the severe shortening of chromosome ends in the *trf4Δ* mutant. In fact, our results do not provide any straight evidence of a direct link between the extent of subtelomeric RNA accumulation and the severity of telomere shortening. Rather it emerged that the telomeric phenotype of the *trf4Δ* mutant can in part reflect imbalances in the expression level between the protein subunits Est1p, Est2p, Est3p, and the RNA component *TLC1* of the telomerase. We propose that Trf4p stimulates the exosome-mediated degradation of *TLC1* through a polyadenylation-independent mechanism. However, in contrast to what happens in cells defective in *rrp6*, *trf4* deletion causes only high levels of *TLC1*, whereas the expression of *EST1*, *EST2*, and *EST3* remains unchanged. It was previously demonstrated that recruitment of the telomerase holoenzyme is mediated by the heterodimeric Ku70/80 complex, which binds the chromosome ends and interacts with the telomerase via a small stem loop region of *TLC1* [42]. Thus, the excess of *TLC1* in the *trf4Δ* mutant could interfere with the recruitment of the telomerase at the chromosome ends and ultimately lead to telomere shortening.

**Figure 7:**
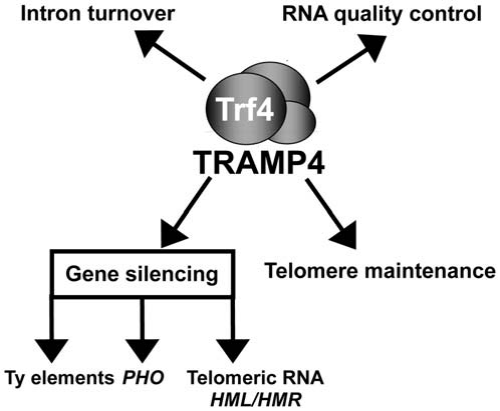
Combined view on the current knowledge of the different roles of the TRAMP4 complex. *PHO*; mRNAs encoding proteins involved in phosphate metabolism.

To conclude, in this work we provide experimental evidence demonstrating that in addition to RNA surveillance, Trf4p and Trf5p participate in post-transcriptional regulatory networks that connect RNA degradation with DNA metabolism and gene regulation (Figure 7). Although the polyadenylation activity of Trf4p clearly enhances the efficiency of degradation of a broad variety of RNAs via the TRAMP4/exosome complex, expression of the Trf4 protein rather than its polyadenylation activity emerged to be essential for the maintenance of effective post-transcriptional regulatory pathways in the cell.

## Materials and Methods

### Yeast Strains and Plasmid Construction

Manipulations of *S. cerevisiae* strains were performed by standard procedures. Cells were grown in YPD (1% yeast extract, 2% peptone, 2% glucose), YPGal (1% yeast extract, 2% peptone, 2% galactose), or in synthetic minimal medium (0.67% Bacto-yeast nitrogen base without amino acid, 2% glucose, and amino acid supplements as required) at 30°C. Temperature shifts to 37°C were done in a shaking water bath. Yeast strains are described in Table S5. The *trf5Δ* and *rrp6Δ* mutant strains were purchased from Open Biosystems. Replacement of the *trf5* and the *rrp6* genes with the *kanMX6* cassette was confirmed by PCR following the manufacturer's instructions. The strain with C-terminal TAP-fusion of *FPR1* (*YNL135C*) was purchased from BioCat (Heidelberg, Germany). The correct integration of the TAP-tag was verified by PCR. The *trf4Δ* strain is a derivative of BY4741 in which the *trf4* gene was replaced with the *natMX4* marker by homologous recombination as previously reported [52]; primer sequences can be provided upon request.

To complement *trf4Δ* with the wild type allele of *trf4* the coding region of *trf4* was PCR amplified from *S. cerevisiae* BY4741 genomic DNA with primers XmaI-trf4-Fw (5′-GTCCCGGGAAATATGGGGGCAAAGAGTGTAAC-3′) and trf4-Rev-SalI (5′-ACGTCGACTTATTAAAGGGTATAAGGATTATAT-3′) (restriction sites are underlined). The insert was cloned in pGEMT-easy vector (Promega), digested with XmaI and SalI restriction enzymes and ligated into the same sites on the pNOPPATA1L vector to generate pNOPPATA1L(*pNOP1::TRF4*). *Trf4Δ* cells were transformed with pNOPPATA1L(*pNOP1::TRF4*) and transformants were selected for leucine prototrophy in synthetic medium at 30°C. The correct integration of the fragment was verified by sequencing. Control *trf4Δ*/pNOPPAT1L and BY4741/ pNOPPATA1L strains were selected for leucine prototrophy onto synthetic medium after transformation of *trf4Δ* and BY4741 cells with the pNOPPAT1L plasmid.

To express *trf4* or *trf5* from an inducible pGAL1 promoter the coding regions of *trf4* and *trf5* were PCR amplified from *S. cerevisiae* BY4741 genomic DNA with primers SacI-trf4-Fw (5′-GTGAGCTCAAATATGGGGGCAAAGAGTGTAAC-3′) and trf4-Rev-XhoI (5′ACCTCGAGTTATTAAAGGGTATAAGGATTATAT-3′) or BamHI-trf5-Fw (5′-AAGGATCCCATAATGACAAGGCTCAAAGCAAAATA-3′) and trf5-Rev-XhoI (5′-AGCTCGAGTTATTAAAGAGCCTGGCCTTTAGAG-3′). The fragments were cloned into pGEMT-easy vector, digested with *Sac*I-*Xho*I or with *BamH*I-*Xho*I and ligated into the same sites of pYC6/CT (Invitrogen) to generate pSAL1 (*pGAL1::TRF4*) or pSAL2 (*pGAL1::TRF5*), respectively. *trf4Δ trf5Δ* mutant cells complemented with wild type *TRF4* by the pRS416-*TRF4* plasmid were successively transformed with either pSAL1 or pSAL2. Transformants were selected onto YPD supplemented with blasticidin (InvivoGen) 150 µg/ml at 30°C. To induce the loss of the pRS416-*TRF4* plasmid, blasticidin resistant colonies were inoculated three times on synthetic medium supplemented with 2% galactose, blasticidin (15 µg/ml) and 5-fluoro-orotic acid (1 µg/ml; Zymo Research) at 30°C. Transformation with pSAL1 or pSAL2 and loss of pRS416-*TRF4* was confirmed by restriction digestion with *Sac*I-*Xho*I or with *BamH*I-*Xho*I of plasmid DNA preparations purified from clones that were uracil auxotrophic, blasticidin resistant, and glucose sensitive.

### RNA Purification

Total RNA was extracted with the hot phenol extraction method. Single yeast colonies were inoculated in 5 ml YPD or YPGal medium supplemented with the appropriate amount of selective drug (G418, 200 µg/ml; clonNat, 100 µg/ml) and incubated overnight at 30°C (pre-cultures). Pre-cultures were diluted to an OD_600_ of 0.1 in 50 ml of fresh YPD or YPGal medium without drugs and grown at 30°C to an OD_600_ of 0.7. Cells were collected by centrifugation for 5 min at 3,000 g and resuspended in AE buffer (50 mM Na-acetate, 10 mM EDTA, pH 5.3) with 1% SDS. After adding one volume of phenol (pH 5.3), the suspension was vigorously shaken for 1 min and incubated at 65°C for 4 min in a heating block (Thermomixer comfort, Eppendorf). The aqueous phase was separated from the phenol phase by centrifugation at 20,000 g and extracted again with phenol (pH 5.3) and then with chloroform. Total RNA was precipitated from the aqueous phase by the addition of 2.6 volumes of ice-cold ethanol and of 1/10 volume of 1 M Na-acetate (pH 5.3) for 20 min on ice. The precipitated RNA was recovered by centrifugation at 20,000 g for 30 min at 4°C, the pellet was washed with 70% ethanol and resuspended in DEPC-treated water.

To remove contaminating DNA, RNA preparations were treated with DNA-*free*™ (Ambion) according to the manufacturer's instructions. The integrity of RNA samples was routinely checked by gel electrophoresis (1.2% agarose - 6% formaldehyde) in 1× HEPES buffer (50 mM HEPES [pH 7.8], 10 mM EDTA) and RNA was quantified by UV-spectrophotometry (A_260_) with a Nanodrop device (Witeg AG).

### *In Vivo* Depletion of Trf4 or Trf5 Proteins

Single colonies of the *trf4Δ trf5Δ/*pSAL1 or of the *trf4*Δ *trf5Δ/*pSAL2 mutant strains were inoculated in YPGal liquid medium supplemented with blasticidin (150 µg/ml; InvivoGen) at 30°C and grown overnight. The following day, cultures were diluted to an OD_600_ of 0.1 in 300 ml of fresh YPGal medium and grown at 30°C to an OD_600_ of 0.7. Cultures were equally split into three tubes and cells were collected by centrifugation at RT for 5 min at 3,000 g. After centrifugation, one-third of the culture was rapidly frozen in liquid nitrogen and stored at −70°C (*t_0_* time point); one-third was resuspended in YPGal and incubated at 30°C for 1 h (*t_1_* Gal time point), while the rest of the culture was inoculated in YPD at 30°C for 1 h (*t_1_* Glc time point). Cells were collected by centrifugation and stored at −70°C. Total RNA was purified from the cell pellet as described above.

### Microarray Analysis

DNA microarrays contained 10,944 oligo probes (70-mers) from the Array-Ready Oligo Set Version 1.1 representing 6,388 *S. cerevisiae* ORF*s*, and the Yeast Brown Lab Oligo Extension Version (YBOX vers. 1.0) with 3,456 probes to detect ncRNAs, rRNA precursors, introns, exon-intron and exon-exon junctions, other sequences predicted to be expressed, additional probes for genes with high cross-hybridization potential and controls for array quality measurements and normalization. Details of oligonucleotide selection and probe sequences are available from the Operon website (www.operon.com). All microarray data are available at the Stanford Microarray Database (SMD) or at the Gene Expression Omnibus (GEO) with accession number GSE16107.

The probes were printed on epoxy coated glass slides (Nexterion slide E) at the Center for Integrative Genomics, University of Lausanne, Switzerland. Oligo arrays were blocked in 5× SSC, 0.1 mg/ ml BSA, 0.1% SDS for 1 h at 42°C, and subsequently washed three times in 0.1× SSC for 5 min at RT, rinsed in water for 30 s, and dried by centrifugation (500 g for 2 min). The slides were used the same day.

Microarray analysis was performed by competitive hybridization of Cy3 and Cy5 fluorescently labeled cDNA. Total RNA (25 µg) was reverse transcribed with SuperScript RT II (Invitrogen) in the presence of 2.5 mM aminoallyl-dUTP (Sigma) and dNTPs, with a 1∶1 mixture of dT20V and random nonamer (N9) primers (5 µg of each, Sigma). After first strand cDNA synthesis, RNA was hydrolyzed with 0.1 M NaOH and 0.1 M EDTA at 65°C for 15 min, and samples were neutralized with 0.35 M HEPES (pH 8.0). Clean up of the reaction mix was performed in Microcon-YM30 (Millipore) filled with distilled water. Amino-allyl containing cDNA was eluted with 100 mM NaHCO_3_ (pH 9.0) and covalently linked to either fluorescent Cy3 or Cy5 NHS-monoester (GE Healthcare). Thereby, cDNAs derived from wild-type control cells were labeled with Cy3, the ones derived from mutant cells with Cy5. Unincorporated dyes were removed with the QIAquick PCR Purification Kit (Qiagen). The samples were mixed in standard formamide based hybridization buffer (Ocimum Biosolution Hybridzation Solution, Cat. No. 1180-000010) supplemented with 1 mg/ml poly(A) in a final volume of 20 µl, and competitively hybridized to yeast oligo arrays in a sealed hybridization chamber (Corning) at 42°C for 12–16 h. Arrays were successively washed in three buffer chambers filled with 2× SSC (300 mM NaCl, 30 mM Na-citrate, pH 7.0), 0.2% SDS; 2× SSC; and 0.2× SSC. The first wash was performed at 42°C for 12 min, the subsequent washes at RT for 12 min. After briefly rinsing in ethanol, microarrays were scanned with an Axon Instruments Scanner 4200A (Molecular Devices). Scanning parameters were adjusted to give similar fluorescent intensities in both channels. Data were collected with GenePix Pro 5.1 (Molecular Devices) and spots with abnormal morphology were excluded from further analysis. Array data were exported to Acuity 4.0 (Molecular Devices) and normalized to the mean of ratio of medians = 1 excluding the signals from control features. We collected three biological replicates each for determining the relative changes of transcript levels in the *trf4Δ*, *trf5Δ* and *trf4Δ*/*TRF4-DADA* mutant cells compared to the respective wild-type cells (total of 9 arrays). Data were filtered in Acuity for regression correlation (Rgn^2^>0.5), signal to noise ratio >2.5 in both channels, and only features that met these criteria in >60% of arrays were considered for further analysis (total 7481 features; Dataset S1). Data were exported into Microsoft Excel to determine percentile ranks and to perform SAM (version 3.0 [28]). We used the web interface for Cyber-T (http://cybert.microarray.ics.uci.edu/) to employ statistical analyses based on regularized *t*-tests that use a Bayesian estimate of the variance among gene measurements within an experiment [34].

The 715 unique features (9.5% of all analyzed features) that were on average at least 2-fold changed with an FDR<5% in either the *trf4Δ*, *trf5Δ*, or *trf4Δ/TRF4-DADA* replicates were compiled (Table S1). The genes and arrays were hierarchically clustered based on Pearson correlations with Cluster 3.0 [53] and the result was visualized as a heatmap with Java TreeView 1.0. [54] (Figure 1A). Commonly enriched GO terms among list of genes were retrieved with GO Term Finder that uses a hypergeometric distribution with Multiple Hypothesis Correction (i.e., Bonferroni Correction) to calculate *p*-values (SGD; www.yeastgenome.org).

### Quantitative Real-Time PCR

qRT-PCR was performed with an ABI Prism 7000 Sequence Detection System (ABI Prism) and the Power SYBR Green PCR Master Mix (Applied Biosystems) according to the manufacturer's instructions. The first strand cDNA was synthesized with 5 µg of total RNA, 50 µM oligo(dT)_20_, 0.1 µM random hexamers and SuperScript RT III (Invitrogen). RNA was subsequently hydrolyzed with 125 mM NaOH and 10 mM EDTA at 65°C for 15 min. The mix was neutralized with 400 mM Tris-HCl (pH 8.0) and loaded onto a Microcon-YM30 (Millipore) concentrator column filled with 10 mM Tris-HCl (pH 8.0). After centrifugation for 8 min at 13,500 g the microcon was filled again with 10 mM Tris-HCl (pH 8.0). This step was repeated twice. After elution, the cDNA was used as template for PCR with the following conditions: 95°C for 10 min; 40 cycles at 95°C for 15 s, and 60°C for 1 min. Transcript abundance was calculated as log_2_ of normalized ratios with the Pfaffel method of relative quantification [55]. Data were normalized to actin mRNA levels. (All primers used for quantitative real time PCR analysis and for strand specific reverse transcription are listed in Table S6.) Primer sequences for the strand specific synthesis of the antisense-Ty1 (RTL) cDNA and of the antisense-PHO5, -PHO11, and -PHO89 cDNAs can be provided upon request.

### *In Vivo* Cross-Linking and Ribonucleoprotein-Immunopurification-Chip Analysis (X-RIP-Chip)

1 L of fresh YPD medium was inoculated with an overnight pre-culture of yeast cells (OD_600_ = 0.1) that were further cultured at 30°C to an OD_600_ = 0.7. RNAs and proteins were cross-linked with 1% formaldehyde that was added directly to the culture for 7 min at RT. The cross-linking reaction was subsequently quenched by the addition of 125 mM glycine (pH 7.0) for 5 min at RT. During cross-linking and quenching reactions the cultures were maintained under constant shaking at 100 rpm. Cells were harvested by centrifugation (1,500 g) at 4°C, washed twice with 50 ml of ice-cold PBS, and lysed in 5 ml of ice-cold lysis buffer (20 mM Tris-HCl [pH 7.5], 140 mM KCl, 1.8 mM MgCl_2_, 0.1% NP-40, 0.1 mM DTT, 10% glycerol, 0.2 mg/ml heparin, 0.5 µg/ml Leupeptin, 0.8 µg/ml Pepstatin, 50 U/ml SUPERaseIn [Amersham]) by grinding in mortar filled with liquid nitrogen. Contaminating DNA was digested with 20 U/ml RNAse-free DNase I (Promega). The WCE was centrifuged twice at 13,000 g for 10 min at 4°C to remove cell debris. The protein concentration of the extract was determined with the Bradford method (Bio-Rad Protein Assay, BioRad). To purify total RNA from extracts for microarray analysis (input RNA sample), 100 µl of WCE was digested with Proteinase K (0.4 mg/ml) for 30 min at 37°C, heated up to 70°C for 45 min to reverse the formaldehyde cross-linking, and proteins were extracted with phenol-chloroform-isoamyl alcohol (PCI, 25∶24∶1). RNA was precipitated with 1.5 M LiCl at −20°C overnight and collected by 30 min centrifugation (14,000 g) at 4°C. The RNA pellet was washed twice with 70% ethanol and resuspended in DEPC-treated water.

Cross-linked TAP-tagged proteins were captured from the WCE as follows: 300 µl rabbit IgG-coupled agarose beads (Sigma) were equilibrated at 4°C in lysis buffer supplemented with 5% BSA. WCE was added to the blocked IgG beads and mixed on a rotator overnight at 4°C. TAP-tagged proteins were then recovered by spinning down the IgG beads at 72 g for 2 min at 4°C. Beads were thoroughly washed three times with ice-cold lysis buffer supplemented with increasing concentrations of NaCl (100 mM, 200 mM, and 350 mM). RNP complexes were digested in 1 ml of elution buffer (50 mM Tris-HCl [pH 7.0], 1% SDS, 5 mM EDTA, 10 mM DTT, 140 mM KCl, 5% glycerol, 0.01% NP-40) with 100 µl Proteinase K (4 mg/ml) for 20 min at 37°C. Formaldehyde cross-linking was reversed by incubation of the eluate at 70°C for 45 min in a gently shaken heating block. Immunopurified RNA (IP-RNA) was isolated by extraction with PCI and isopropanol precipitation. The RNA pellet was washed twice with 70% ethanol and resuspended in DEPC-water.

For the microarray analysis of the IP-RNA, 5 µg of total RNA (input RNA) and 500 ng of IP-RNA were converted into Cy3 and Cy5 fluorescently labeled cDNA, respectively, and samples were competitively hybridized on yeast oligo arrays as described above. We collected data from three biological replicates with Trf4-TAP, from two replicates with Fpr1-TAP and from one untagged control (BY4741 strain) sample. Array data were filtered in Acuity for signal to noise ratio >3 for the channel with the input RNA (Cy3), and percentile ranks for filtered data were calculated based upon the log_2_ of the Cy5/Cy3 ratio in each experiment with Excel (Dataset S2).

### Northern Blot Analysis

Northern blotting experiments were performed as previously described [56],[57]. Briefly, 35 µg of total RNA in RNA loading buffer (50% formamide, 6% formaldehyde, 50 mM HEPES [pH 7.8], 0.25% xylene cyanol, 0.25% bromophenol blue, 10% glycerol) was loaded on a 1.5% agarose-6% formaldehyde gel and fractionated in 1× HEPES buffer (50 mM HEPES [pH 7.8]; 10 mM EDTA) at 50–60 Volts for 15 h. After washing of the gel in distilled water for 15 min, the RNA was partially cleaved with 75 mM NaOH for 15 min. The gel was neutralized in a solution comprised of 5 M Tris-HCl (pH 7.0) and 1.5 M NaCl for 15 min, and equilibrated in 10× SSC for 20 min. Capillary transfer of the RNA to Hybond-N+ membranes (Amersham) was performed in 10× SSC over night. RNA was UV-crosslinked to the membrane in a UV Stratalinker 1800 (Stratagene) with 1200 µJ.

DNA probes for hybridization were prepared by random incorporation of α-[^32^P] dATP with the Random Prime DNA Labeling Kit (Roche). Unincorporated α-[^32^P] dATP was removed by MicroSpinTM G-25 Columns (GE Healthcare). DNA templates for the preparation of the randomly labeled probes were produced by PCR amplification with primer pairs rps24A-int-Fw (5′-AGAAATGGTATGTTAAAAAGTGCTCAGATG-3′) and rps24A-int-Rev (5′-CAGCGTCAGACTGAGAAAAAAC-3′) or rpl2B-int-Fw (5′-CGCATAATTATGGCAAATGTTATGAAGG-3′) and rpl2B-int-Rev (5′-CGAATAACTCTACCTGTTTAAATGAGG-3′) to detect the *RPS24A* and *RPL2B* introns, and primer pairs rps24A-ex-Fw (5′-TCTGACGCTGTTACTATCCGTACTA-3′) and rps24A-ex-Rev (5′-AATCGGCGTTACGACGAGCAACCT-3′) or rpl2B-ex-Fw (5′-CACACCAGATTAAGACAAGGTGCT-3′) and rpl2B-ex-Rev (5′-GAACCACGTAGTAAACCGGTTCTTCT-3′) to detect the *RPS24A* or *RPL2B* mRNAs. The DNA template for the preparation of the randomly labeled *PGK1* probe was previously described [56]. Hybridization was carried out in rolling tubes in hybridization buffer containing 50% formamide, 5× SSPE (750 mM NaCl, 50 mM NaH_2_PO_4_, 5 mM EDTA, pH 7.4), 5× Denhardts solution, 1% SDS, and 200 µg/ml salmon sperm DNA according to standard procedures. The result of the hybridization was visualized with a Phosphor Imager.

### Western and Dot Blot Analysis of Immunopurified Protein Complexes

Two µl of protein samples were directly spotted onto the nitrocellulose membrane (Whatman) for dot blot analysis. For Western blots, proteins were separated on 12% polyacrylamide gels, transferred to nitrocellulose membranes, and incubated with antibodies indicated in the figure legends. The anti-TAP antibodies were previously described [5]. To generate anti-Air2 antibodies a C-terminal fragment of Air2p (comprising amino acids 210–344 of Air2p) was cloned in pET22b (Novagen) and expressed in the *Escherichia coli* strain BL21. The resulting C-terminal Air2p fragment contained a [His]_6_ tag fusion on its C-terminus. The protein was expressed in LB medium according to the manufacturer (Novagen) and affinity purified on Ni^2+^-NTA agarose (Sigma) under denaturing conditions as described [5]. After further purification on reverse phase chromatography (GE Healthcare) in FPLC, approximately 100 µg of the purified protein was used for three injections into a rabbit (Eurogentec). Peroxidase-conjugated swine anti-rabbit antibodies (DAKO) served as secondary antibodies for detection of the primary antibodies with the ECL Plus Western blotting detection system (Amersham).

### Telomere Length Analysis

Telomere length measurement was carried out as previously described [44]. Genomic DNA was prepared from yeast cells grown in YPD and according to standard procedures. DNA from each strain was digested overnight with the restriction enzyme *Xho*I and fractionated by 1% agarose gel electrophoresis in 1× TBE buffer (90 mM Tris-borate, 2 mM EDTA) at 40 Volts for 15 h. DNA was transferred to a Hybond-N+ membrane (Amersham) and Southern blot was performed by hybridization with a telomeric probe (26G; 5′-TGTGGGTGTGGTGTGTGGGTGTGGTG-3′) that was end-labeled with γ-[^32^P] ATP and T4 polynucleotide kinase (Biolabs). All hybridizations were done in 200 mM Na_2_HPO_4_, 1 mM EDTA, 2% SDS, 1% BSA and 50 µg/ml salmon sperm DNA. The average telomeric length for each lane was estimated by a 1 kb DNA ladder (peqLab) that was run in a lane next to the *Xho*I digested genomic DNA. The ladder was probed with the same DNA ladder after γ-^32^P end-labeling.

## Supporting Information

Figure S1 Classes of RNAs that accumulate in *trf4Δ*, *trf5Δ*, and *trf4Δ/TRF4-DADA* mutants. Pie chart classifying the transcripts with more than 2-fold (FDRs<5%) increased expression in in the *trf4Δ* (A), the *trf5Δ* (B), and the *trf4Δ/TRF4-DADA* (C) mutant strains as determined by microarray analysis. Microarrays contained 10,944 oligo probes (70-mers) representing 6,388 *S. cerevisiae* ORFs and 3,456 probes to detect ncRNAs (e.g. snRNAs/snoRNAs), rRNA precursors, INTs, Ty1 retrotransposon elements, exon-intron and exon-exon junctions, and 242 IGRs/CUTs. We infer that the fraction of CUTs and ncRNAs in the three mutants is underestimated as our microarrays do not fully cover all the genome's intergenic regions (including both strands) as well as antisense RNAs.
(2.19 MB TIF)

Figure S2 Expression profiles of SnoRNAs and IGRs (CUTs) in RNA surveillance mutants. Microarray analysis of *trf4Δ*, *trf5Δ*, and *trf4Δ/TRF4-DADA* (*DADA*) mutants showing relative changes for a sample of snoRNAs (14 out of 27; *SNR*) and IGRs/CUTs the steady-state levels of which were >2-fold increased (FDRs<5%) in *trf4Δ* mutant cells. snoRNAs and IGRs/CUTs strongly accumulated in the *trf4Δ* mutant, but not in the *trf5Δ* mutant. In addition, most of the snoRNAs (*SNR10*, *SNR11*, *SNR65*, *SNR3*, *SNR72*, *SNR45*, *SNR48*, and *SNR49*) and some IGRs (*IGR67*, *IGR130*) still showed an increase of more than 1.5-fold relative to WT cells upon overexpression of Trf4-DADA in *trf4Δ* mutant cells. Microarrays are the same as shown in Figure 1.
(1.66 MB TIF)

Figure S3 Retrotransposon Ty1 elements accumulate in the *trf4Δ* Mutant and are restored to wt levels by Trf4p-DADA overexpression. (A) Bar diagram representing relative changes of Ty1 retrotransposon RNAs as found by microarray analysis of the *trf4Δ*, the *trf5Δ* and the *trf4Δ/TRF4-DADA* (*DADA*) mutants. The values are averages of the levels of Ty1 retrotransposon transcripts as displayed by 68 out of 96 Ty1 retrotransposon probes showing more than 2-fold increase (FDR<5%) in the *trf4Δ* mutant. Microarrays are the same as shown in Figure 1. (B) Bar diagrams show the results of the qRT-PCR analysis for the Ty1 retrotransposon elements in RNA surveillance mutants (*trf4Δ*, *trf4Δ/TRF4-DADA*, *trf5Δ*, and *rrp6Δ*). The scheme above the bar diagram represents the Ty1 retrotransposon locus: grey arrow (UP) indicates the position of the UP-αTy1 primer used for strand specific synthesis of *anti-sense-Ty1* (*RTL*) cDNA; convergent solid arrows (αTyF and αTyR) indicate the primer pairs anti-Ty-Fw and anti-Ty-Rv used for the quantification of the *RTL* cDNA; convergent grey arrowheads (TyF and TyR) show the location of Ty-Fw and Ty-Rv primers used for the quantification of the *TyA/B* cDNA. Consistent with the microarray analysis the expression of Ty1 retrotransposon is restored to WT levels by the overexpression of Trf4p-DADA in *trf4Δ* mutant cells. RNA amounts were normalized to *ACT1* mRNA and are compared to the isogenic WT strain. Relative changes of transcript abundances (log_2_ ratio scale) represent averages from two independent qRT-PCR analyses. The RNA was also used for the microarray analysis presented in Figure 1A.
(1.39 MB TIF)

Figure S4 Intron expression profiles in RNA surveillance mutants. Bar diagrams show the results of qRT-PCR analysis for a group of introns (*RPS9A-INT*; *RPL16A-INT*, *RPL7B-INT1*, *GCR1-INT*, and *RPL40A-INT*) in RNA surveillance mutants (*trf4Δ*, *trf4Δ/TRF4-DADA*, *trf5Δ*, and *rrp6Δ*). qRT-PCR analysis was performed with intron-specific primers. Overexpression of Trf4p-DADA in *trf4Δ* mutant cells abolished the accumulation of the first intron of *RPL7B* (*RPL7B-INT1*) and of *GCR1* (*GCR1-INT*) and reduced by 3.6-fold (log_2_) the abundance of the intron of *RPS9A* (*RPS9A-INT*). RNA amounts were normalized to *ACT1* mRNA and are compared relative to the isogenic wild-type strain. Relative changes of transcript abundances (log_2_ ratio scale) represent averages from two independent qRT-PCR analyses. The RNA was also used for the microarray analysis presented in Figure 1A.
(0.76 MB TIF)

Figure S5 Expression profiles of transcripts derived from the silenced *HML/HMR* cassettes and of genes involved in chromatin silencing. (A) Bar diagram representing the results of the qRT–PCR analysis for *HMLα1* and *ARS318* in RNA surveillance mutants (*trf4Δ*, *trf4Δ/TRF4-DADA*, *trf5Δ*, and *rrp6Δ*). Whereas overexpression of Trf4p-DADA restored the abundance of *HML*α*1* to WT levels, *ARS318* transcripts still exhibited a 2-fold increase in *trf4Δ/TRF4-DADA* mutant cells. Both *HML*α*1* and *ARS318* RNAs strongly accumulated in the *rrp6Δ* mutant strain. RNA levels were normalized to *ACT1* mRNA and compared to the relative expression in isogenic wild-type strain. Relative changes of transcript levels (log_2_ ratio scale) correspond to the average from two independent experiments. The RNA was also used for the microarray analysis presented in Figure 1A. (B) Bar diagram representing the levels of *SIR2*, *SIR3*, *SIR4*, *SAS5*, and *MCM10* mRNAs in the *trf4Δ* and the *trf5Δ* mutant strains quantified qRT-PCR. RNA amounts were normalized to *ACT1* mRNA and are compared relative to the isogenic WT strain. Relative changes of transcript abundances (log_2_ ratio scale) represent averages from two independent qRT–PCR analyses. The same RNA was used for the microarray analysis shown in Figure 1A.
(1.14 MB TIF)

Figure S6 Expression profiles of *PHO5*, *PHO89*, *anti-PHO5*, and *anti-PHO89* RNAs in RNA surveillance mutants. Strand-specific qRT–PCR analysis examining the steady-state levels of *PHO5*, *anti-PHO5* (A), *PHO89*, and *anti-PHO89* (B) RNAs in RNA surveillance mutants (*trf4Δ, trf4Δ/TRF4-DADA*, *trf5Δ*, and *rrp6Δ*). RNA amounts were normalized to *ACT1* mRNA and compared relative to the isogenic wild-type strain. Relative changes of transcript abundances (log_2_ ratio scale) represent averages from two independent qRT–PCR analyses. The RNA was also used for the microarray analysis presented in Figure 1A.
(1.15 MB TIF)

Table S1 List of genes with 2-fold altered expression (FDR<5%) by deletion of either *trf4* or *trf5* or by overexpression of Trf4p-DADA in the *trf4Δ* mutant strain. Columns indicate the following (from left to right): Clone ID; gene name; systematic name; Probe sequence on the array (70-mer); GO annotations for biological process, function, and cellular compartment; Operon description of gene product; average log_2_ ratio in *trf4Δ* mutants; average log_2_ ratios in *trf4Δ/TRF4-DADA* mutants; average log_2_ ratio in *trf5Δ* mutants; FDRs *trf4Δ*; *p*-value *trf4Δ*; FDR *trf4Δ/TRF4-DADA*; *p*-value *trf4Δ/TRF4-DADA* microarrays; FDR *trf5Δ*; *p*-value *trf5Δ*; *p*-value *trf4Δ* vs. *trf4Δ/TRF4-DADA*; cyberT test of *trf4Δ* vs. *trf5Δ* microarrays; FDR *trf4Δ* vs. *trf4Δ/TRF4-DADA*; FDR *trf4Δ* vs. *trf5Δ* microarrays.
(5.12 MB XLS)

Table S2 Statistics of the 2-fold changed features. Columns indicate the following (from left to right): mutant strain; number of features exhibiting 2-fold up- or downregulation; number of features exhibiting 2-fold up- or downregulation after CyberT test (*p*<0.05); number of features exhibiting 2-fold up- or downregulation after SAM analysis (FDR<0.05%).
(0.02 MB XLS)

Table S3 Significantly enriched GO terms among transcripts that are significantly increased in the *trf4Δ* and the *trf5Δ* mutants. Columns indicate the following (from left to right): Mutant strain; category; gene ontology (GO) term; number of genes with annotations; number of genes in the genome with annotation; *p*-value.
(0.02 MB XLS)

Table S4 Percentile ranks of introns from X-RIP-Chip data. Columns are the same as for Dataset S2. Changes of relative expression (average log_2_ ratios) in *trf4Δ* and *trf5Δ* mutants are shown in separate columns (values are taken from Dataset S1).
(2.57 MB XLS)

Table S5 Yeast strains used in this work. Columns indicate the following (from left to right): *S. cerevisiae* strain; genotype; reference.
(0.03 MB XLS)

Table S6 Oligonucleotide primer sequences for qRT–PCR analyses. Columns indicate the following (from left to right): Oligonucleotide name; target RNA; oligonucleotide sequence in the 5′ to 3′ direction.
(0.03 MB XLS)

Dataset S1 Normalized data from DNA microarray experiments with *trf4Δ*, *trf5Δ*, and *trf4Δ/TRF4-DADA* mutants. Columns indicate the following (from left to right): Oligo ID (Operon); gene name; systematic name; yeast ORF (compatible with SGD); GO annotations for process, function, and cellular compartment; Operon description of the gene product; log_2_ ratio *trf4Δ* mutants (triplicates); average log_2_ ratio *trf4Δ* mutants; log_2_ ratio *trf4Δ/TRF4-DADA* mutants (triplicates); average log_2_ ratio *trf4Δ/TRF4-DADA* mutants; log_2_ ratio *trf5Δ* mutants (triplicates), average log_2_ ratio *trf5Δ* mutants; FDRs *trf4Δ* microarrays; *p*-values *trf4Δ* microarrays; FDRs *trf4Δ/TRF4-DADA* microarrays; *p*-values *trf4Δ/TRF4-DADA* microarrays; FDRs of *trf5Δ* microarrays; *p*-values *trf5Δ* microarrays; *p*-values *trf4Δ* vs. *trf4Δ/TRF4-DADA* microarrays; *p*-values *trf4Δ* vs. *trf5Δ* microarrays; FDRs *trf4Δ* vs. *trf4Δ/TRF4-DADA* microarrays; FDRs *trf4Δ* vs. *trf5Δ* microarrays. An annotation key to the oligos and a comparison of IGRs to recently mapped CUTs [18],[19] is given in separate worksheets.
(4.36 MB XLS)

Dataset S2 Percentile ranks from X-RIP-Chip data. Columns indicate the following (from left to right): Oligo ID (Operon); gene name; yeast ORF (compatible with SGD); GO annotations for process, function, and cellular compartment; Operon description of the gene product; percentile rank in Trf4 affinity isolations (3 biological replicates); average percentile rank of Trf4 affinity isolations; percentile rank of Fpr1 control affinity isolations (2 biological replicates); percentile rank of WT mock control isolations (BY4741); average percentile rank of control isolates (Fpr1, BY4741).
(1.99 MB XLS)
