# Supplementary figures and images for: Distinct Roles of Non-Canonical Poly(A) Polymerases in RNA Metabolism
Source: PLoS Genet. 2009 Jul 10;5(7):e1000555. doi: 10.1371/journal.pgen.1000555 (PMC2700272; doi:10.1371/journal.pgen.1000555)

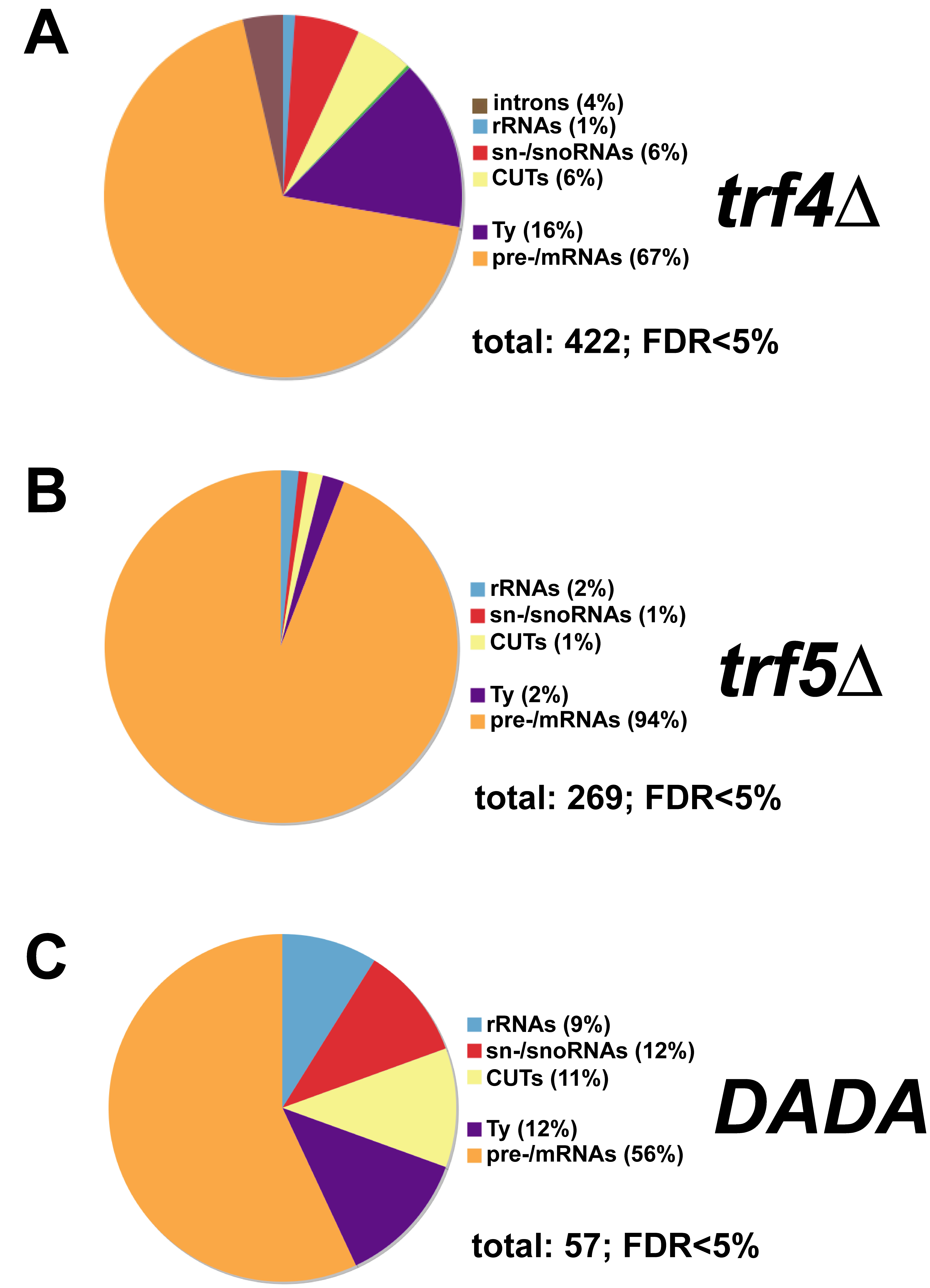

Supplement: Figure S1 — Classes of RNAs that accumulate in trf4Δ, trf5Δ, and trf4Δ/TRF4-DADA mutants. Pie chart classifying the transcripts with more than 2-fold (FDRs<5%) increased expression in in the trf4Δ (A), the trf5Δ (B), and the trf4Δ/TRF4-DADA (C) mutant strains as determined by microarray analysis. Microarrays contained 10,944 oligo probes (70-mers) representing 6,388 S. cerevisiae ORFs and 3,456 probes to detect ncRNAs (e.g. snRNAs/snoRNAs), rRNA precursors, INTs, Ty1 retrotransposon elements, exon-intron and exon-exon junctions, and 242 IGRs/CUTs. We infer that the fraction of CUTs and ncRNAs in the three mutants is underestimated as our microarrays do not fully cover all the genome's intergenic regions (including both strands) as well as antisense RNAs. (2.19 MB TIF) [file pgen.1000555.s001.tif]

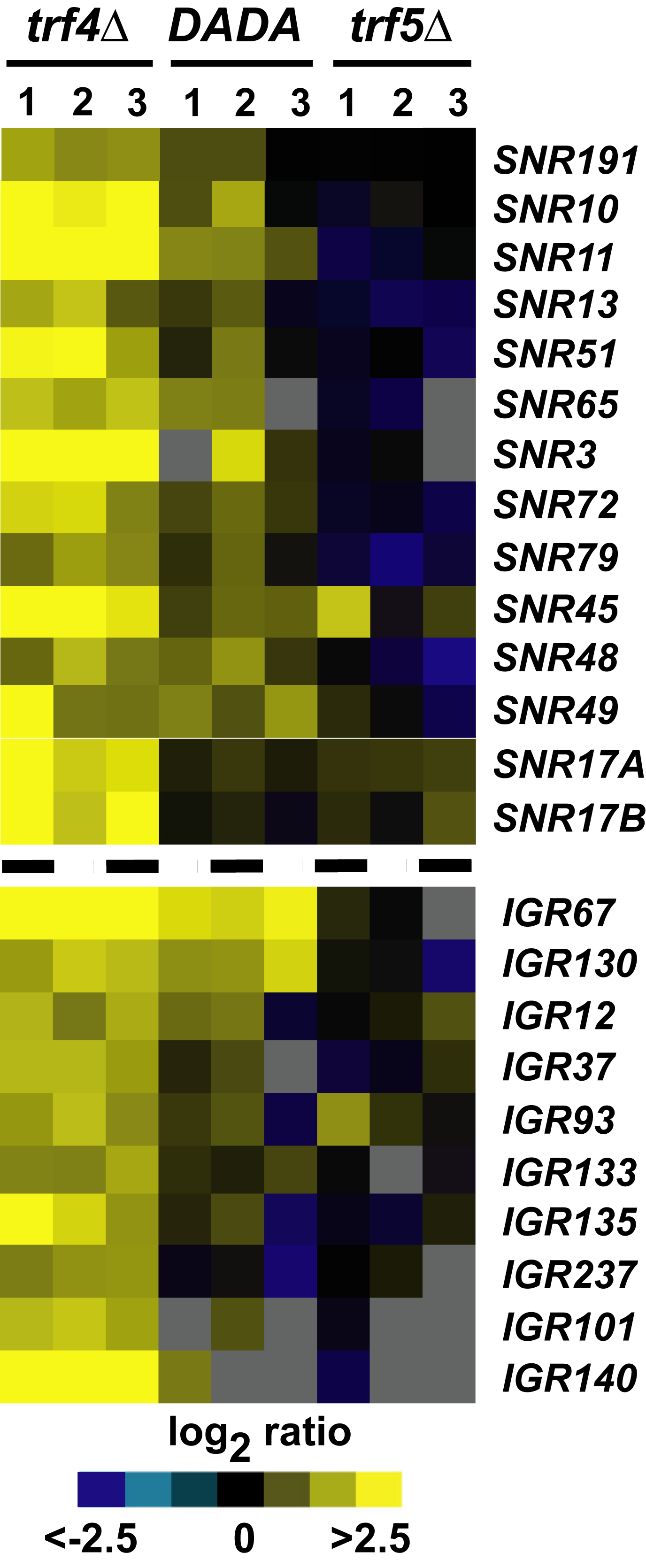

Supplement: Figure S2 — Expression profiles of SnoRNAs and IGRs (CUTs) in RNA surveillance mutants. Microarray analysis of trf4Δ, trf5Δ, and trf4Δ/TRF4-DADA (DADA) mutants showing relative changes for a sample of snoRNAs (14 out of 27; SNR) and IGRs/CUTs the steady-state levels of which were >2-fold increased (FDRs<5%) in trf4Δ mutant cells. snoRNAs and IGRs/CUTs strongly accumulated in the trf4Δ mutant, but not in the trf5Δ mutant. In addition, most of the snoRNAs (SNR10, SNR11, SNR65, SNR3, SNR72, SNR45, SNR48, and SNR49) and some IGRs (IGR67, IGR130) still showed an increase of more than 1.5-fold relative to WT cells upon overexpression of Trf4-DADA in trf4Δ mutant cells. Microarrays are the same as shown in Figure 1. (1.66 MB TIF) [file pgen.1000555.s002.tif]

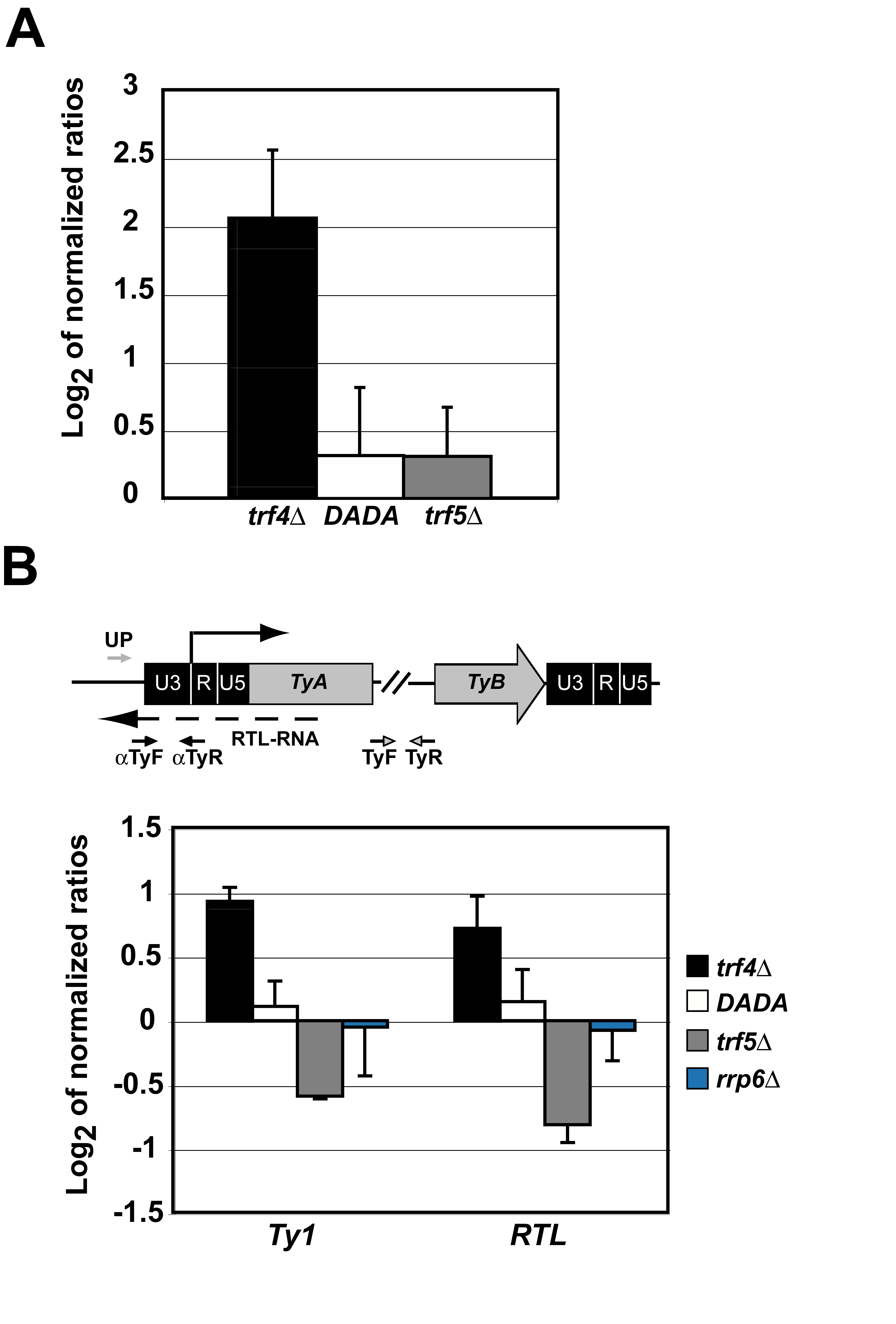

Supplement: Figure S3 — Retrotransposon Ty1 elements accumulate in the trf4Δ Mutant and are restored to wt levels by Trf4p-DADA overexpression. (A) Bar diagram representing relative changes of Ty1 retrotransposon RNAs as found by microarray analysis of the trf4Δ, the trf5Δ and the trf4Δ/TRF4-DADA (DADA) mutants. The values are averages of the levels of Ty1 retrotransposon transcripts as displayed by 68 out of 96 Ty1 retrotransposon probes showing more than 2-fold increase (FDR<5%) in the trf4Δ mutant. Microarrays are the same as shown in Figure 1. (B) Bar diagrams show the results of the qRT-PCR analysis for the Ty1 retrotransposon elements in RNA surveillance mutants (trf4Δ, trf4Δ/TRF4-DADA, trf5Δ, and rrp6Δ). The scheme above the bar diagram represents the Ty1 retrotransposon locus: grey arrow (UP) indicates the position of the UP-αTy1 primer used for strand specific synthesis of anti-sense-Ty1 (RTL) cDNA; convergent solid arrows (αTyF and αTyR) indicate the primer pairs anti-Ty-Fw and anti-Ty-Rv used for the quantification of the RTL cDNA; convergent grey arrowheads (TyF and TyR) show the location of Ty-Fw and Ty-Rv primers used for the quantification of the TyA/B cDNA. Consistent with the microarray analysis the expression of Ty1 retrotransposon is restored to WT levels by the overexpression of Trf4p-DADA in trf4Δ mutant cells. RNA amounts were normalized to ACT1 mRNA and are compared to the isogenic WT strain. Relative changes of transcript abundances (log2 ratio scale) represent averages from two independent qRT-PCR analyses. The RNA was also used for the microarray analysis presented in Figure 1A. (1.39 MB TIF) [file pgen.1000555.s003.tif]

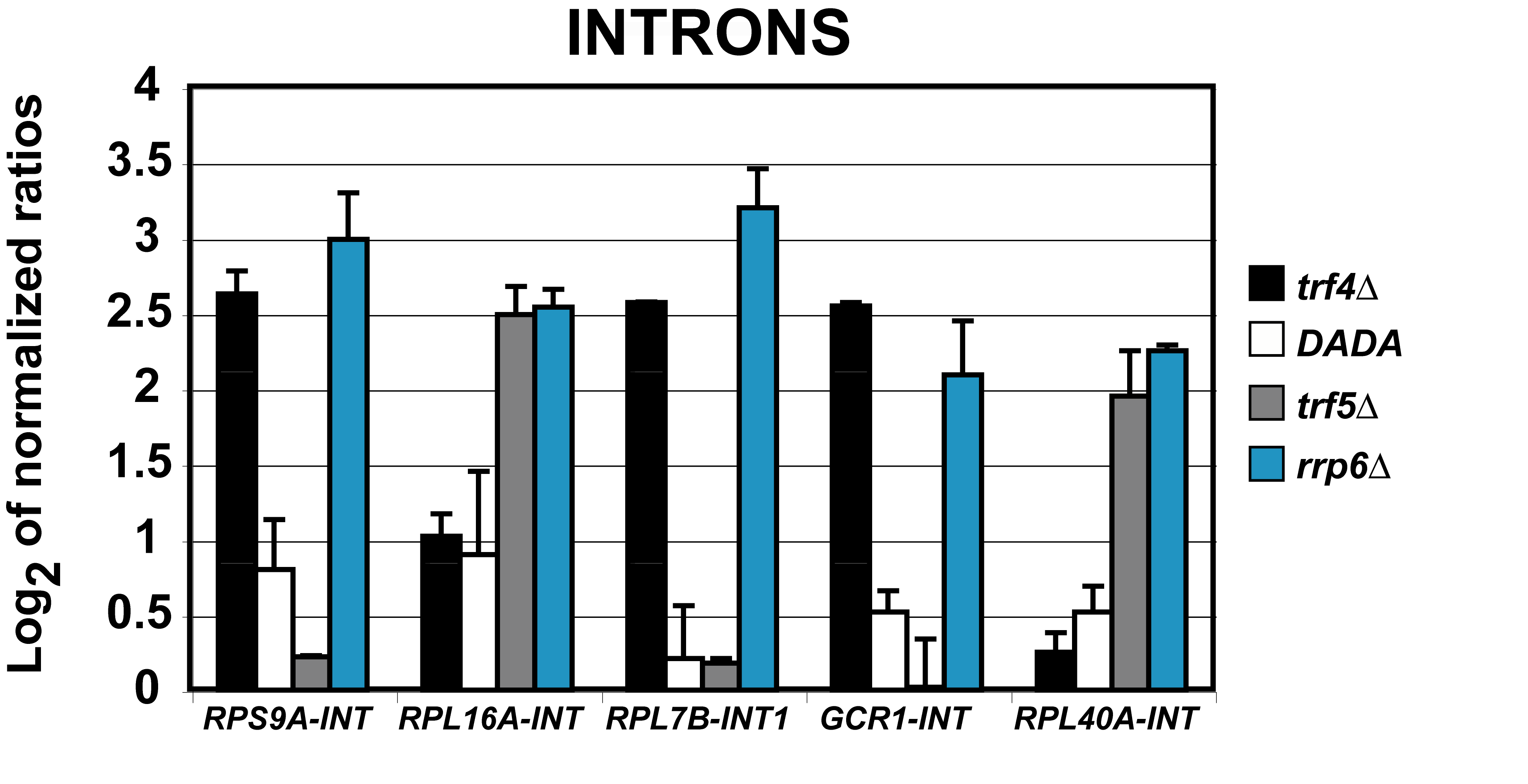

Supplement: Figure S4 — Intron expression profiles in RNA surveillance mutants. Bar diagrams show the results of qRT-PCR analysis for a group of introns (RPS9A-INT; RPL16A-INT, RPL7B-INT1, GCR1-INT, and RPL40A-INT) in RNA surveillance mutants (trf4Δ, trf4Δ/TRF4-DADA, trf5Δ, and rrp6Δ). qRT-PCR analysis was performed with intron-specific primers. Overexpression of Trf4p-DADA in trf4Δ mutant cells abolished the accumulation of the first intron of RPL7B (RPL7B-INT1) and of GCR1 (GCR1-INT) and reduced by 3.6-fold (log2) the abundance of the intron of RPS9A (RPS9A-INT). RNA amounts were normalized to ACT1 mRNA and are compared relative to the isogenic wild-type strain. Relative changes of transcript abundances (log2 ratio scale) represent averages from two independent qRT-PCR analyses. The RNA was also used for the microarray analysis presented in Figure 1A. (0.76 MB TIF) [file pgen.1000555.s004.tif]

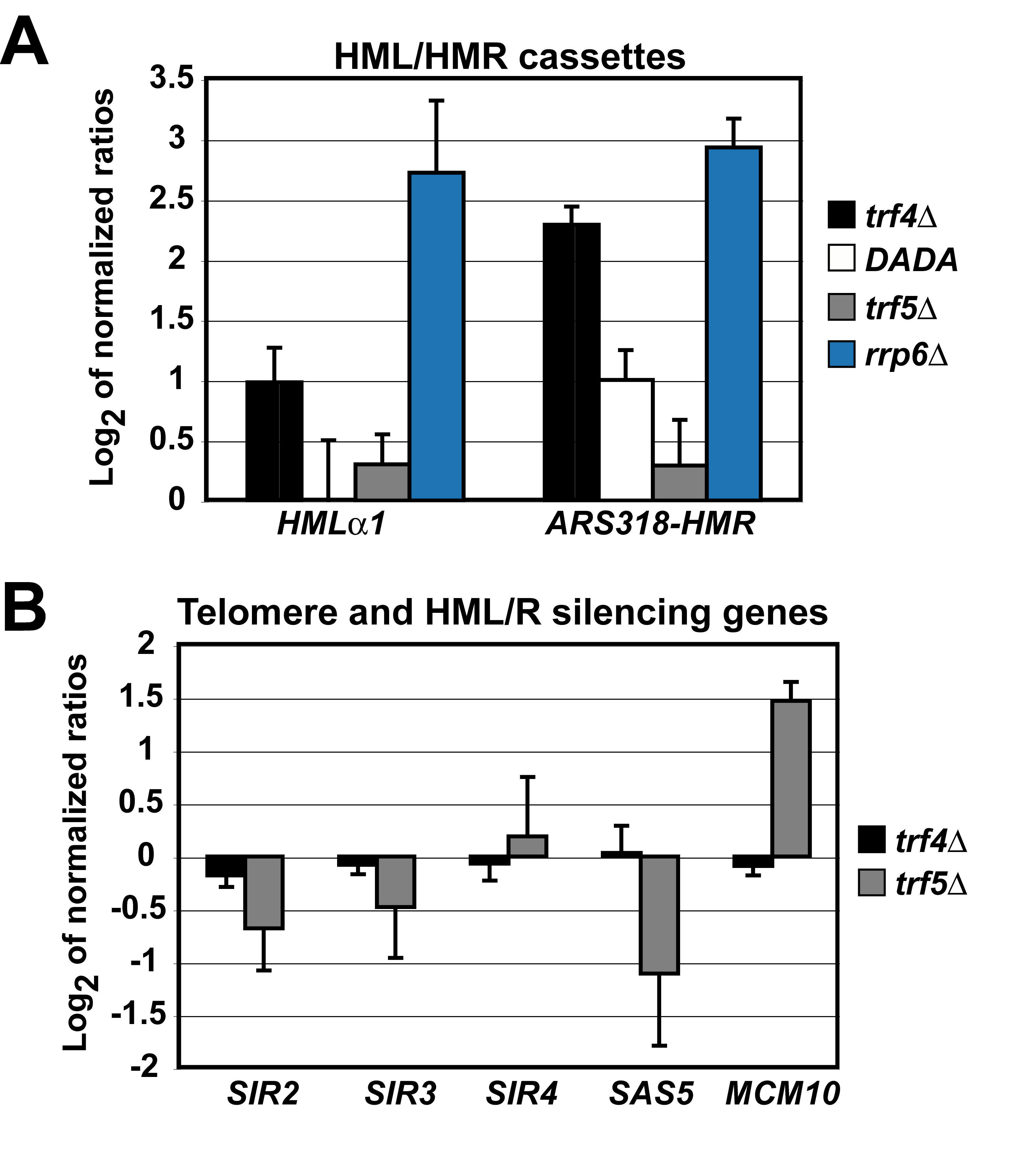

Supplement: Figure S5 — Expression profiles of transcripts derived from the silenced HML/HMR cassettes and of genes involved in chromatin silencing. (A) Bar diagram representing the results of the qRT–PCR analysis for HMLα1 and ARS318 in RNA surveillance mutants (trf4Δ, trf4Δ/TRF4-DADA, trf5Δ, and rrp6Δ). Whereas overexpression of Trf4p-DADA restored the abundance of HMLα1 to WT levels, ARS318 transcripts still exhibited a 2-fold increase in trf4Δ/TRF4-DADA mutant cells. Both HMLα1 and ARS318 RNAs strongly accumulated in the rrp6Δ mutant strain. RNA levels were normalized to ACT1 mRNA and compared to the relative expression in isogenic wild-type strain. Relative changes of transcript levels (log2 ratio scale) correspond to the average from two independent experiments. The RNA was also used for the microarray analysis presented in Figure 1A. (B) Bar diagram representing the levels of SIR2, SIR3, SIR4, SAS5, and MCM10 mRNAs in the trf4Δ and the trf5Δ mutant strains quantified qRT-PCR. RNA amounts were normalized to ACT1 mRNA and are compared relative to the isogenic WT strain. Relative changes of transcript abundances (log2 ratio scale) represent averages from two independent qRT–PCR analyses. The same RNA was used for the microarray analysis shown in Figure 1A. (1.14 MB TIF) [file pgen.1000555.s005.tif]

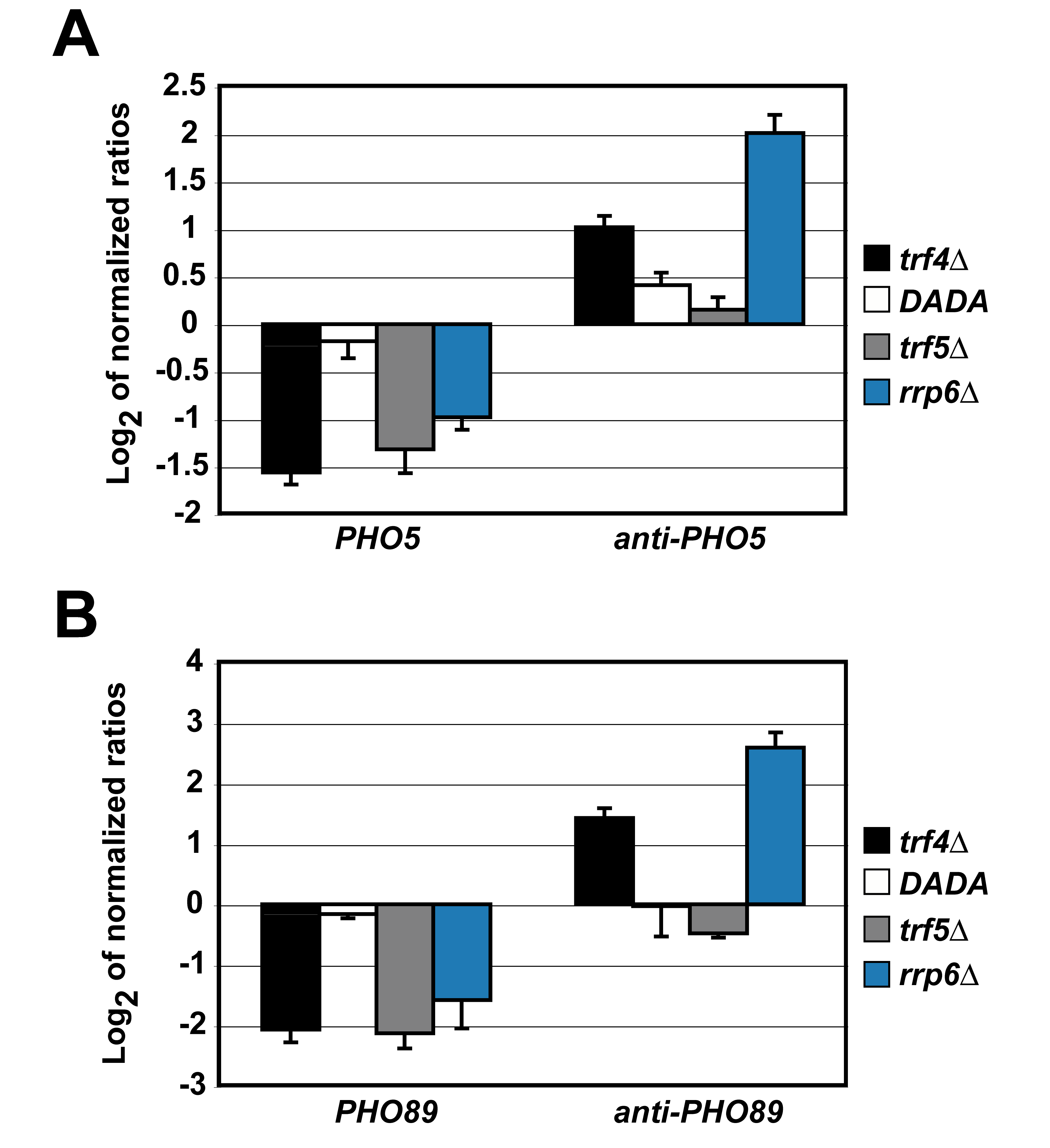

Supplement: Figure S6 — Expression profiles of PHO5, PHO89, anti-PHO5, and anti-PHO89 RNAs in RNA surveillance mutants. Strand-specific qRT–PCR analysis examining the steady-state levels of PHO5, anti-PHO5 (A), PHO89, and anti-PHO89 (B) RNAs in RNA surveillance mutants (trf4Δ, trf4Δ/TRF4-DADA, trf5Δ, and rrp6Δ). RNA amounts were normalized to ACT1 mRNA and compared relative to the isogenic wild-type strain. Relative changes of transcript abundances (log2 ratio scale) represent averages from two independent qRT–PCR analyses. The RNA was also used for the microarray analysis presented in Figure 1A. (1.15 MB TIF) [file pgen.1000555.s006.tif]
